# Probing Electrostatic and Hydrophobic Associative Interactions in Cells

**DOI:** 10.1021/acs.jpcb.4c05990

**Published:** 2024-10-30

**Authors:** Weiyan Zuo, Meng-Ruo Huang, Fabian Schmitz, Arnold J. Boersma

**Affiliations:** 1 Cellular Protein Chemistry, Bijvoet Centre for Biomolecular Research, Faculty of Science, Utrecht University, Utrecht 3584 CH, The Netherlands; 2 DWI-Leibniz Institute for Interactive Materials, Aachen 52074, Germany; 3 Institute of Technical and Macromolecular Chemistry, RWTH Aachen University, Aachen 52074, Germany

## Abstract

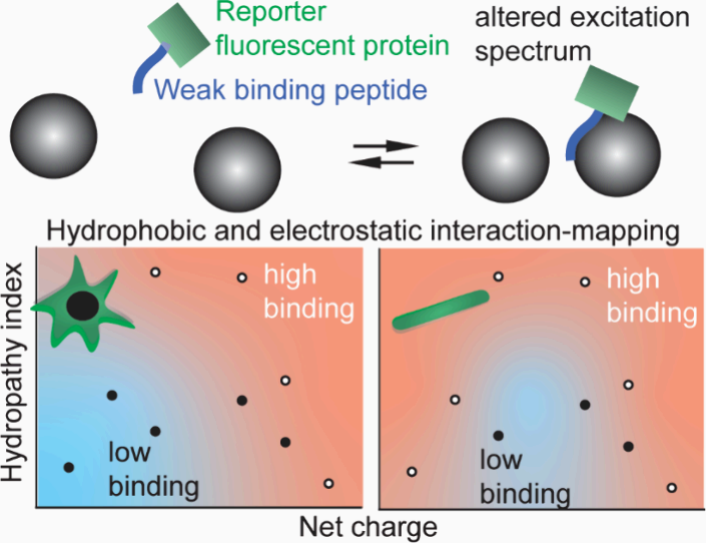

Weak nonspecific interactions between biomacromolecules determine the cytoplasmic organization. Despite their importance, it is challenging to determine these interactions in the intracellular dense and heterogeneous mixture of biomacromolecules. Here, we develop a method to indicate electrostatic and hydrophobic associative interactions and map these interactions. The method relies on a genetically encoded probe containing a sensing peptide and a circularly permuted green fluorescent protein that provides a ratiometric readout. Inside bacterial and mammalian cells, we see that the cytoplasmic components interact strongly with cationic and hydrophobic probes but not with neutral hydrophilic probes, which remain inert. The *Escherichia coli* cytoplasm interacts strongly with highly negatively charged hydrophilic probes, but the HEK293T cytoplasm does not. These associative interactions are modulated by ATP depletion. Hence, the nonspecific associative interaction profile in cells is condition- and species-dependent.

## Introduction

The intracellular environment has a high chemical diversity consisting of proteins, polynucleotides, lipids, glycans, ions, water, and metabolites. The concentration of macromolecules is between 100 and 400 g/L,^1−4^ and the large surface area presented by the molecules and their chemical diversity give rise to nonspecific and nonfunctional interactions due to steric, electrostatic, van der Waals, hydrogen bonding, and hydrophobic interactions.^5^ The nonspecific associative interactions, also termed stickiness, counterbalance the repulsive effects of the volume excluded by crowders, which are of steric origin.^6,7^ The stickiness that a protein experiences is species-dependent, as each has a different proteome composition and density.^8,9^ Weak nonspecific associative interactions are characterized by low specificity and dissociation constants in the high micromolar to millimolar range.^10,11^ More specific interactions require precise tuning of the protein interfaces to overcome the relatively low concentration of the binding partner surface area.^12,13^

The stickiness of proteins has major implications as it affects protein distribution and destabilizes individual proteins.^14,15^ In addition, misfolded proteins become sticky by exposing hydrophobic residues that recruit chaperones through attractive interactions with low specificity. Next to protein stability^16^ and folding,^17^ these attractive interactions slow down diffusion.^18^ In the negatively charged proteome, the hydrophilicity and the negative charge of the protein surfaces increase the folding stability,^19,20^ and an increase in the positive charge and hydrophobicity of a test molecule decreases its diffusion through enhanced stickiness.^21−24^ Even single-point mutations can affect the stability of native proteins by changing interactions with the environment.^14^ Moreover, these interactions depend on cellular conditions. For example, drugs that induce cytoskeletal depolymerization increased stickiness in a protein-specific manner.^22^ Hence, nonspecific electrostatic and hydrophobic interactions are important in cells and govern protein behavior.

A few methods exist to determine nonspecific interactions in the cells. Mass spectrometry combined with cross-linking or pull-down assays, either in-cell or in cell lysates,^25^ provides common binding partners such as chaperones, cytoskeletal proteins, and polynucleotide-binding proteins. While these measurements provide binding partners, obtaining thermodynamic data from them is challenging. In-cell thermodynamic data can be extracted from in-cell NMR of protein stability or diffusion, fluorescence-based methods such as Förster resonance energy transfer (FRET) to determine protein conformation or interaction,^11,26,27^ or diffusion measurements with fluorescence recovery after photobleaching (FRAP), fluorescence correlation spectroscopy (FCS), or single-particle tracking.^28,29^ While these methods provide the effect of environmental stickiness on protein folding, diffusion, and binding, they are each hampered by specific drawbacks, such as the need for high sensor concentration or sensitivity to parameters other than binding. Therefore, a method to quantify nonspecific interactions that can be used in cells is highly needed.

Here, we present genetically encoded probes for identifying nonspecific associative interactions of dense in vitro and in-cell solutions. The ratiometric fluorescence excitation sensors consist of short peptides with systematically varied charges and hydrophobicity. These are linked to a circularly permuted GFP that provides the readout. We verified associative interactions with protein-crowded solutions to show the effect of the protein concentration on nonspecific binding. We then compared the readouts with HEK293T and *Escherichia coli* (*E. coli*) cells to find qualitative similarities with crowded BSA solutions, except for the negatively charged probes, which appear more inert in HEK293T than in *E. coli* or crowded BSA solutions.

## Methods

### Expression and Purification of the Probes

The synthetic gene encoding each probe was purchased from Integrated DNA Technologies (IDT, Coralville, USA) and cloned into a pRSET A plasmid between restriction sites NdeI and *Hin*dIII. The *E. coli* strain BL21 (DE3) was transformed with the plasmid. The cells were grown to an OD_600_ of 0.6–0.8 in AN LB medium (10 g/L NaCl, 10 g/L tryptone, and 5 g/L yeast extract) with 1 mg/mL ampicillin at 37 °C, after which gene expression was induced overnight with 0.1 mM isopropyl-β-d-thiogalactoside (IPTG) at 25 °C. The cells were spun down, and the pellet was resuspended in a buffer (10 mM sodium phosphate (NaPi), 100 mM NaCl, and 0.1 mM phenylmethylsulfonyl fluoride, pH 7.4) and lysed by a Multi Cycle Cell Disrupter (Constant Systems Limited, Northants, UK). The cell debris was removed by centrifugation (12,000 rpm). The supernatant was supplemented with 10 mM imidazole and purified by immobilized metal affinity chromatography (His-trap FF column, Cytiva); wash/elution buffer: 20/250 mM imidazole, 50 mM NaPi, and 300 mM NaCl, pH 8.0. The sensor was further purified by Superdex 200 10/300 GL size-exclusion chromatography (Cytiva) in 10 mM NaPi, pH 7.4. The purified sensor was treated with tobacco etch virus (TEV) protease for 3 h at 30 °C to remove the His-tag. Fractions that did not bind to the His-trap FF column were collected. The expression and purification were analyzed by 15% SDS-PAGE, and the bands were visualized by Coomassie blue staining. Fractions containing pure protein were aliquoted and stored at −80 °C.

### BSA (−74e)

To a solution of BSA (Sigma-Aldrich, >98%, lyophilized powder; 1.0 g, 15 μmol) in a 0.05 M phosphate buffer (100 mL, pH = 7.5) was slowly added succinic anhydride (354 mg, 3 mmol) at 0 °C. The pH of the solution was maintained in the range of 7.5–8.0 by adding 0.5 M sodium hydroxide solution. Upon dissolution, the solution was agitated with magnetic stirring at 4 °C for 30 min. The solution was then concentrated by ultrafiltration centrifugal tubes (Vivaspin, 30,000 MWCO, 20 mL) at 4 °C to give the modified BSA. The modified lysine residues were quantified by using MALDI-TOF mass spectrometry. In comparison to BSA, the succinic-BSA conjugate showed a 2800 Da molecular weight increase, which means that approximately 28 lysine groups were modified.

### BSA (+92e)

To a solution of BSA (300 mg, 4.5 μmol) and EDCI·HCl (50 mg, 0.26 mmol) in water (15 mL) was added ethylenediamine (10% aqueous, 1 mL, 1.5 mmol) at room temperature. The pH of the solution was maintained in the range of 4.5–5.0 by adding 1 N hydrochloric acid. The solution was left at room temperature for 2 h, after which a second batch of EDCI·HCl (25 mg, 0.13 mmol) was added, and the pH of the solution was readjusted to 4.5–5.0. The mixture was left at room temperature for 18 h and then concentrated by ultrafiltration centrifugal tubes (Vivaspin, 30,000 MWCO, 20 mL) at 4 °C to give the modified BSA. The modified carboxylic acids were quantified by using MALDI-TOF mass spectrometry. In comparison to BSA, the ethylenediamine-BSA conjugate showed a 2517 Da molecular weight increase, which means that approximately 55 carboxylic acids were modified.

### DNA Titration

DNA solution (salmon sperm DNA, Invitrogen, Waltham, MA, USA) was diluted to the desired concentration with a NaPi buffer (10 mM, pH 7.4). A 100 μL solution with the desired DNA concentration and the purified probe was placed in a quartz cuvette, and its fluorescence excitation spectrum at 520 nm emission was recorded on a Horiba Fluoromax-4P spectrometer at 25 °C. The DNA fluorescence background spectrum from DNA solution without a probe was recorded separately and subtracted.

### BSA Titration

Wild-type (Sigma-Aldrich, St. Louis, USA) and surface-modified BSA samples were dissolved in a NaPi buffer (10 mM NaPi and 100 mM KCl, pH 7.4) to the desired concentration. We purified BSA by concentrating a 50 mg/mL stock solution to 200 mg/mL using an Amicon 10 kD cutoff filter. The concentrate was diluted again with a fresh buffer, and this procedure was repeated four times to yield a 150 mg/mL BSA stock solution. One hundred μL solutions containing the probe (1 μM) and BSA at the desired concentrations were placed in a 96-well plate and mixed thoroughly by a pipet. The plate was scanned immediately by a SpectraMax iD3 microplate reader (Molecular Devices, San Jose, USA). Each sample was measured in random order with equilibration up to 40 min. The excitation spectra at 520 nm emission were recorded at room temperature. The corresponding background spectrum from BSA solutions without probes was subtracted. We assume that BSA binds not more than one peptide in its hydrophobic cleft at a time. The ratio (intensity_405nm_/intensity_488nm_) was plotted against BSA concentrations. Subsequently, the data were fitted to a Hill function: *y* = ratio_START_ + (ratio_END_ – ratio_START_) × *x^n^*/(*k^n^* + *x^n^*), where *k* is the dissociation constant (*K*_D_) and *n* is the Hill coefficient.^30^

### Circular Dichroism (CD) Measurements

The CD spectra of BSA (−17e), BSA (−74e), and BSA (+92e) were recorded by using a J-1500 CD spectrometer (JASCO, Easton, USA). The protein concentration was 0.1 mg/mL (1.5 μM). The following parameters were used: CD scale 200 mdeg/1.0 dOD; D.I.T. 2 s; bandwidth 1 nm. All measurements were three independent repeats.

### Transfection of HEK293T Cells

HEK293T cells were cultured in a DMEM medium containing 10% fetal bovine serum (FBS) and 1% penicillin–streptomycin (P/S) at 37 °C with 5% CO_2_. For transfection, cells were seeded into 8-well chamber slides (Ibidi, Gräfelfing, Germany), with 6.7 × 10^4^ cells in 200 μL of the growth medium (DMEM+10% FBA+1% P/S) for each well. The synthetic gene encoding each probe was purchased from IDT and cloned into a pcDNA3.1 plasmid (between restriction sites *Nhe*I and *Bam*HI). One day after plating, cells were transfected using Lipofectamine 2000 (Thermo Fisher, Waltham, USA) according to the manufacturer’s instructions. One μL of Lipofectamine and 250 ng of the pcDNA3.1 plasmid encoding the probe were diluted with 25 μL of the Opti-MEM I reduced serum medium (Gibco) separately and incubated for 5 min at room temperature. Then, the mixture of diluted DNA and Lipofectamine (50 μL) was incubated at room temperature for 20 min and subsequently added to 150 μL of DMEM (serum-free, antibiotic-free). The cell culture medium in the slide was changed with the transfection medium (200 μL). The transfection medium was exchanged for a complete growth medium after 6 h of incubation and incubated for 24 h before measurement.

### Confocal Fluorescence Microscopy of HEK293T Cells

Before imaging, the cell medium was replaced with fresh DMEM (with HEPES, no phenol red, Gibco). The cells were imaged by a confocal microscope (Leica SP8, Wetzlar, Germany) with a 63×/1.35 water-immersion objective at 37 °C. The probes were excited using a 405 nm LED and a 488 nm argon laser (laser power 10%) separately, and fluorescence signals were recorded between 510 and 550 nm with a PMT detector accordingly.

For the ATP depletion experiment, carbonyl cyanide-*p*-trifluoromethoxyphenylhydrazone (FCCP, Sigma-Aldrich) was dissolved in DMSO (10 mM) and subsequently diluted to 2 μM with a DMEM medium (with HEPES, no phenol red, Gibco). Before imaging, the cell medium was replaced with DMEM (with HEPES, no phenol red, Gibco) containing 2 μM FCCP. It was previously shown that 2 μM FCCP was sufficient to deplete the cells of ATP.^31,32^ Cells were imaged by the same method as described every 5 min.

The fluorescence intensity of the cells was determined in ImageJ. The intensity of the blank cell cytoplasm from each image was set as a background and subtracted from the measured fluorescence intensities. The 405 nm channel intensity was plotted versus the 488 nm channel intensity for each cell. Linear fits with *R*^2^ > 0.99 were obtained with the intercepts set as zero. The slope was taken as the ratio. The ratios from BSA measurements for ratio_bound_ and ratio_unbound_ determination were converted from the plate reader to microscopy measurements by using the formula derived in Figure S13. The percentage of the probe bound inside cells was calculated using percentage bound = |ratio_bound_ – ratio_cell_|/|ratio_bound_ – ratio_unbound_, where ratio_cell_ is the measured ratio in cells.

### Confocal Fluorescence Microscopy of *E. coli* Cells

The *E. coli* strain BL21 (DE3) was transformed with the corresponding plasmid encoding one of the probes (see above). The cells were grown to an OD_600_ of 0.1–0.2 in 5 mL of the MOPS minimal medium^33^ supplemented with 10 mM glucose at 30 °C. Cells without a plasmid that we used for background correction were treated in the same manner. The cells were then induced by 50 μM IPTG at 25 °C for 1 h. Before imaging, cells encoding the corresponding sensor were mixed with control cells, spun down by centrifugation, and resuspended in the MOPS minimal medium. 10 μL of this mixture was added to a coverslip modified with (3-aminopropyl) triethoxysilane (APTES). The cells were imaged by applying the same settings as for HEK293T cell experiments. The fluorescence intensity of the cells was determined in ImageJ. The intensity of cells encoding blank plasmids from each image was set as the background and subtracted from the measured fluorescence intensities. The 405 nm channel intensity was plotted versus the 488 nm channel intensity for each cell. Linear fits with *R*^2^ > 0.99 were obtained with the intercepts set as zero.

### FRAP Measurements

The FRAP measurements were performed with the same microscope. The bleached area was set with a 3.5 μm width, a 3.37 μm height, and a 10.79 μm perimeter for each cell in the cytoplasm and was bleached for 0.18 s with 100% LED power at 405 nm. We used the 405 nm LED to bleach, as this is the main excitation band, with the drawback of incomplete bleaching due to limited LED power. The data are highly reproducible, suggesting the absence of photoswitching. The data were exported from Leica software LAS X. The normalized intensity was plotted versus time, and the data were fitted with a one-phase exponential equation, *f*(*t*) = *a*(1 – e^–*bt*^). The recovery half-time was calculated using τ_1/2_ = ln(0.5)/(−*b*).^34^

## Results

### Probe Interaction with Protein-Crowded Solutions

**Figure 1:**
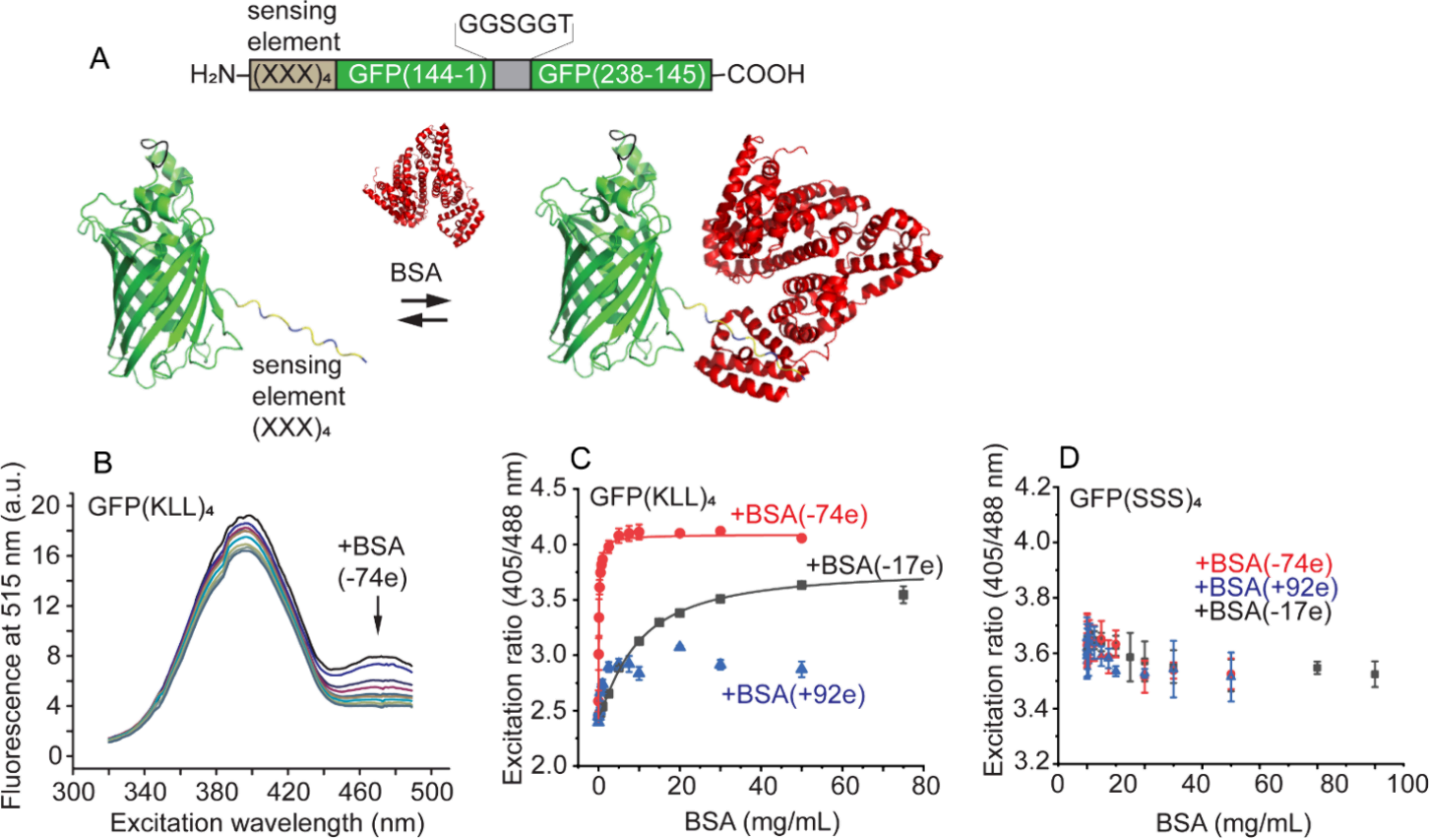
Sensing weak binding with a ratiometric probe with a systematically varied sensing element. (A) Design of the probe for nonspecific interactions and sensing concept. The sensing element is placed at position 144 of a circular permuted GFP, whose fluorescence properties change upon analyte binding to the peptide. BSA from PDB 4f5s. GFP generated with Alphafold2. (B) Excitation spectrum at the emission of 509 nm for the titration of BSA (−74e) to GFP(KLL)_4_, showing relatively increased quenching of the 488 nm excitation band compared to the 405 nm band upon binding. (C) Ratiometric readout of the titration of different modified BSAs to GFP(KLL)_4_, demonstrating the charge dependence on the binding curves. (D) Titration of GFP(SSS)_4_ to the same BSAs showing that a neutral hydrophilic sensing element does not bind. All experiments were performed in triplicate. Error bars are standard deviations over the triplicate.

We first developed a probing method to determine nonspecific associative interactions (Figure 1A and the Supporting Information). We characterized the method for sensing interactions by titrating bovine serum albumin (BSA) to a cationic hydrophobic peptide fused to cpGFP, GFP(KLL)_4_ (Figure 1). BSA is negatively charged (−17e) with a hydrophobic cleft that binds hydrophobic peptides.^35^ We chemically supercharged BSA by modifying 28 Lys residues with succinic acid to obtain BSA (−74e) and modified 55 Glu/Asp residues with ethylene diamine to yield BSA (+92e). Circular dichroism suggests that the main characteristics of the secondary structure of BSA are retained after these modifications (Figure S5).

Titration of wtBSA to GFP(KLL)_4_ increased the 405 nm/488 nm ratio, indicating probe binding (Figure 1B,C). Here, the signal saturated at about 40 mg/mL BSA. Electrostatics increased the binding of BSA (−74e) to GFP(KLL)_4_, saturating already at ∼5 mg/mL BSA (−74e). The GFP(KLL)_4_ does not change in the presence of BSA (+92e). The GFP(SSS)_4_ probe, which is hydrophilic and neutral, did not sense any of the BSAs (Figure 1D), confirming that the BSAs do not associate with the GFP barrel directly. We cannot exclude the binding of BSA to GFP after binding the KLL peptide. The insensitivity of GFP(SSS)_4_ allows its use to identify cpGFP-specific effects in the remainder of the experiments.

**Figure 2:**
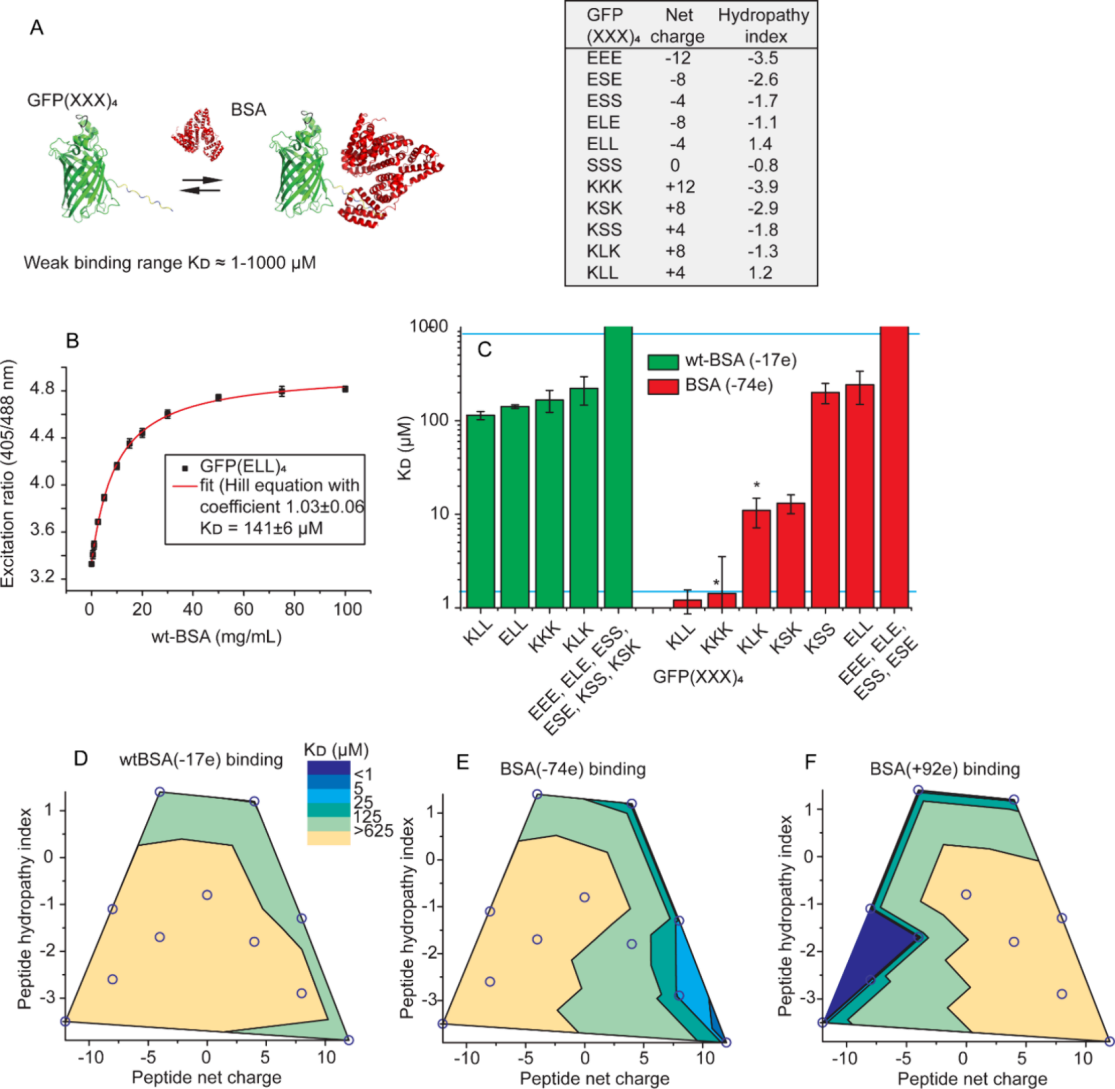
Dependence of the probe binding specificity on the sensing domain physicochemical properties. (A) Domains selected to cover a well-spread range of net charges and hydrophobicities, as expressed with the Kyte–Doolittle scale. (B) Titration of BSA to the ELL probe showing a binding curve. ELL binding saturates at ∼30 mg/mL BSA. (C) Comparison of the determined dissociation constants (*K*_D_) for the different probes for wtBSA (−18e) and BSA (−74e). The blue lines indicate the upper and lower bounds for binding affinity determination due to titration up to 100 mg/mL BSA (2 mM) and the application of the 1 μM probe. Asterisks indicate a Hill coefficient below 1. (D–F) Contour plots of the binding affinities of the probes plotted versus their physicochemical properties for the different BSAs. The hydrophobicity index of the sensing domain was calculated according to the Kyte–Doolittle scale,^36^ and the number of lysine or glutamic acid residues determined the net charge of the sensing domain. Coloring and contouring lines guide the eyes.

To dissect the hydrophobic and electrostatic components of the interaction, we substituted the sensing domain with peptides covering a range of net charges and hydrophobicities (Figure 2A). We selected lysine, glutamate, leucine, and serine as positive, negative, hydrophobic, and hydrophilic amino acids, respectively. We substituted the amino acids in the repeat units systematically where GFP(KKK)_4_ and GFP(EEE)_4_ are most highly charged, GFP(KLL)_4_ and GFP(ELL)_4_ are at the hydrophobic extreme, and GFP(SSS)_4_ is neutral. Hydrophobic GFP(LLL)_4_ could not be overexpressed. The resulting excitation spectra for all 11 purified sensors show the excitation bands at about 400 and 488 nm (Figure S6). The relative 488 nm band intensity followed the order anionic < hydrophilic ∼ no sensing domain < cationic. Thus, the deprotonated form of the fluorophore appears to be stabilized by a cationic sensing element and destabilized by a negative one.

Next, we titrated the three BSAs with all probes (Figure 2). The BSA required additional purification, and the sensing domain required the removal of the His-tag to measure accurate binding data. In most cases, BSA binding increases the excitation ratios for cationic and hydrophobic probes and decreases the excitation ratios for the negative ones (Figures S7–S9). We estimated the dissociation constants using the Hill equation to compare the binding. We find that the cationic and hydrophobic probes saturate with wtBSA (−17e) to give *K*_D_s in the order of 0.1–0.2 mM (Table S1 and Figure 2C). Especially in the case of KLL and ELL, the sensing amplitude is precise, with a 150% ratio change upon BSA binding. The other probes are bound too weakly for *K*_D_ determination.

The electrostatic contribution to binding is pronounced (Figure 2C–F). For example, changing from wtBSA (−17e) to BSA (−74e) reduces the *K*_D_ of GFP(KLL)_4_ binding by >100-fold from the 0.1–0.2 mM range to <2 μM. In addition, while hydrophilic cationic probes KSK and KSS do not detect wtBSA, a *K*_D_ in the 10–200 μM range can be observed with BSA (−74e). As expected, cationic BSA (+92e) binds strongly to negative probes such as GFP(EEE)_4_ with an apparent *K*_D_ = 0.4 ± 1.1 μM. Negatively charged probes bind wtBSA too weakly to determine a binding constant. The strongest binders showed Hill coefficients below one, likely because the dissociation constant is in the same range or lower than the probe concentration in this experiment (1 μM), and the *K*_D_s determined in this range are an upper limit of the actual *K*_D_.

We aimed for quantitative insight into the weaker interacting probes that do not saturate. The surfaces are near under these conditions: At 100 mg/mL BSA, it was estimated that only 23% of BSA is not in physical contact with the other BSAs at a particular time.^37^ We used maximal binding ratios for BSA (+92e) to estimate that at 100 mg/mL wtBSA, GFP(EEE)_4_, GFP(ESS)_4_, and GFP(ESE)_4_ are at least 30–40% bound. The titration of BSA to these probes follows a sigmoidal shape and gives higher Hill coefficients: This could be due to the increased thermodynamic activity of BSA at high concentration (see Supporting Discussion 2). Because ∼2 mM wtBSA does not saturate the probe, the *K*_D_ will be higher than 1 mM. Thus, we can determine dissociation constants between 5 μM and 1 mM and identify binding outside this range.

To compare how the probe hydropathy and net charge determine its binding and specificity, we mapped the binding affinities in a contour plot for the different BSAs (Figure 2D–F). The graphs clearly show that the sensing domain net charge is the main driver for high binding affinities of the hydrophilic probes. This effect is especially notable for supercharged BSAs. Hydrophobic probes bind all three BSAs, while hydrophilic probes do not interact (KLL and ELL vs KSS and ESS). Hence, the propensity of the probe to interact associatively follows the charge and hydrophobicity.

### The Nonspecific Interaction Profile in HEK393T

**Figure 3:**
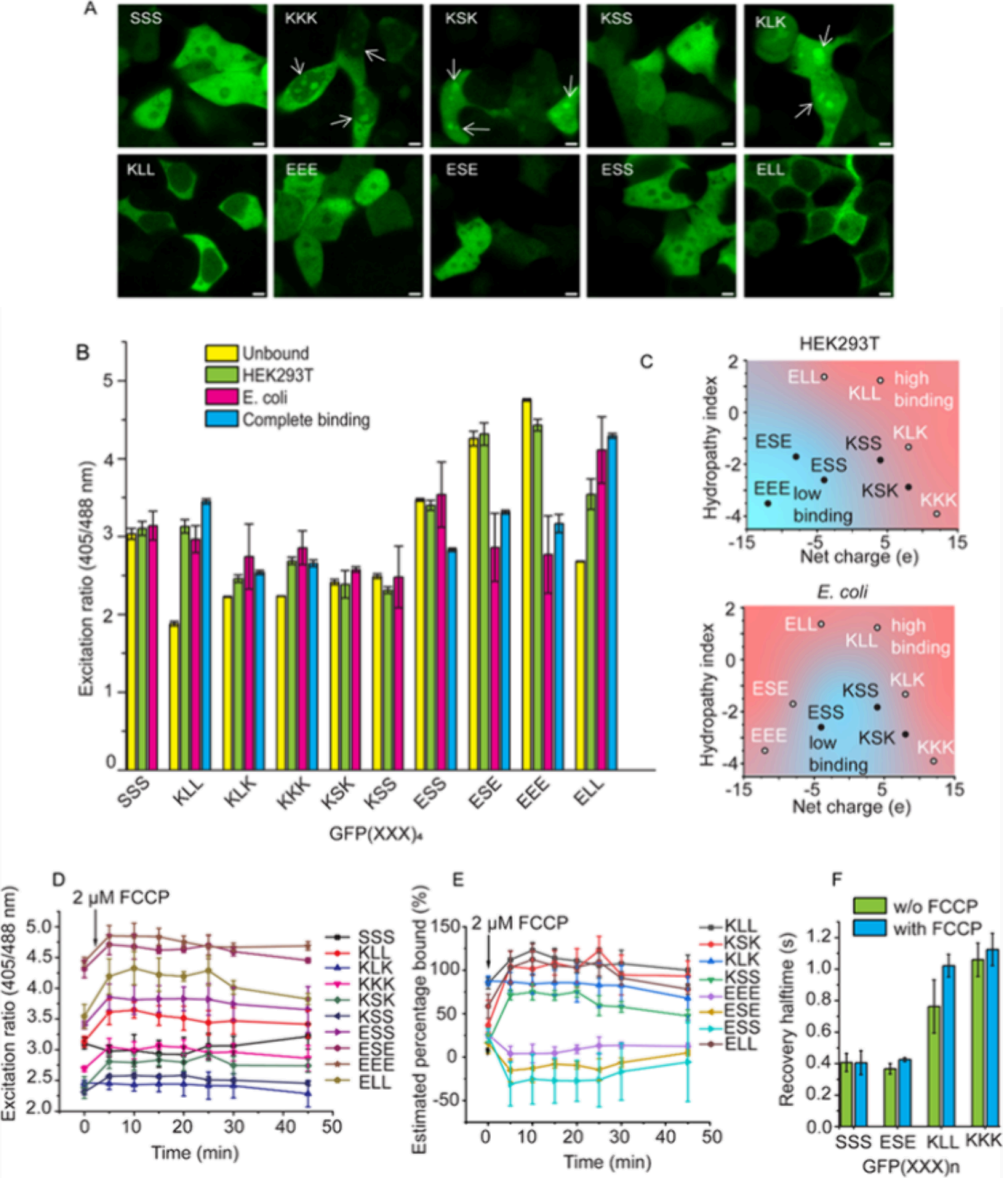
Monitoring the interaction profile of HEK293T and *E. coli*. (A) Representative fluorescence confocal microscopy images of the different probes showing variation in the localization. White arrows indicated the locations of foci. (B) Ratiometric readouts of the different probes in HEK293T and *E. coli* compared to the unbound and fully bound state as obtained from the BSA experiments. The ratios from the BSA experiments were converted from the plate reader to microscopy ratios using the calibration line (Figure S11). (C) Physicochemical properties of the peptides compared to their binding state in HEK293T (top) and *E. coli* (bottom). Red shading indicates strong binding (>60%) and blue weak binding (<20%) as determined from (B). (D) Change in the nonspecific binding profile observed upon FCCP treatment of HEK293T cells. Ratiometric readout followed in time for the different probes. (E) Calculated percentage probe bound after calibration of the in vivo data. (F) Recovery half-time obtained from FRAP for the peptides in HEK293T before and 10 min after FCCP addition. The stronger-binding peptides recover more slowly than the weaker-binding peptides. All experiments are the averages of three independent replicates, and the error bar represents the standard deviation for the replicates.

With the *in vitro* measurement as a reference, we determined the interactions in a human cell line (Figure 3). We expressed the constructs in HEK293T cells and imaged the cells by laser scanning confocal microscopy. Next, we calculated the excitation ratio (405/488 nm) (Figure 3B). No clear dependencies of the ratios on the measured probe concentrations are resolved (inferred from the fluorescence intensity), suggesting that the percentage probe bound is constant (Figure S10).

To interpret the ratios in cells, we made a calibration line to interconvert ratios from the confocal microscope and fluorescence plate reader readouts, using the sensors in a buffer in both the microscope and the plate reader (Figure S11). We compared the in-cell ratios to the converted in vitro ratios for unbound and BSA-bound probes to estimate in-cell binding (Figure 3B). Because the ratios have some dependence on the binding partner identity, we did not pursue precise in-cell quantification. Nevertheless, the general trend is similar in wtBSA solutions, with cationic and hydrophobic probes approaching complete binding. In contrast, no apparent interaction is observed for the hydrophilic anionic and neutral probes (Figure 3B,C). To confirm the binding behavior of these probes, we determined their diffusion by fluorescence recovery after photobleaching (FRAP) (Figure 3F and Figure S12).^34^ It can be expected that diffusion is lower for probes sticking to macromolecules, while the size of their binding partners also contributes strongly. The FRAP measurement provided recovery half-times following GFP(SSS)_4_ ∼ GFP(ESE)_4_ < GFP(KLL)_4_ < GFP(KKK)_4_, in line with our estimates from our ratiometric measurements in cells. We can thus conclude that in the range of our probes, the hydrophobic and electrostatic interaction profiles are qualitatively similar in HEK293T and wtBSA.

The interactions of cationic probes can be explained by the predominantly negative charge of the biomacromolecules in cells.^38^ The strong binding of cationic and hydrophobic probes that we see in cells falls in line with the literature: cationic proteins and peptides diffused slower than their neutral counterparts, and protein folding stability is reduced through nonspecific or quinary hydrophobic and electrostatic interactions with the proteome.^14,20,39^ In cells, the probes likely bind the abundant “CRAPome” consisting of chaperones and cytoskeletal proteins^40^ as well as RNA or ribosomes.^21^ The strong binding of cationic and hydrophobic probes may further be correlated with their toxicity.^41^ As chaperones are known to bind unfolded proteins that expose hydrophobic domains, we assessed if the in-cell binding could be correlated with predicted chaperone binding. Prediction software that estimates peptide binding to chaperone Hsp70 BiP in a buffer (Figure S13)^42^ indeed shows that probe binding in HEK293T follows Hsp70 BiP binding scores. While Hsp70 BiP itself is located in the ER, cytosolic Hsp70 could account at least partially for the binding trends that we see.

We assessed whether the cytosolic interactions display spatial heterogeneity (Figure 3A). Some probes distribute inhomogeneously over the cytoplasm: The hydrophobic GFP(KLL)_4_ and GFP(ELL)_4_ were excluded from the nucleus, and the highly cationic GFP(KSK)_4_, GFP(KLK)_4_, and GFP(KKK)_4_ display foci, possibly through colocalization with polyanionic RNA in the nucleolus.^43^ Although the ratios of these probes depend on the location (Figure S14), these are relatively small differences and may be due to different binding partners. Hence, while the probes remain bound, they colocalize with a particular binding partner, and stickiness remains high despite the location in the cell.

### The Nonspecific Interaction Profile in *E. coli*

*E. coli* maintains a higher biomacromolecule density than HEK293T.^1−4^ Therefore, if the probes interact nonspecifically, we expect to see more associative interactions in *E. coli*. We, therefore, tested all probes in *E. coli* BL21 under optimized expression conditions with a stable fluorescence signal over time. We imaged the bacteria with the same settings as those of the HEK293T cells to compare the excitation ratios (Figure 3B). The bacteria are smaller than HEK293T cells and provide fewer photons; therefore, the noise in the experiments is somewhat larger. The ratios are similar to those of the HEK293T cells, with the notable exception of the more negative GFP(ESE)_4_ and GFP(EEE)_4_, which appear to be in a fully bound state in *E. coli*. The more neutral hydrophilic probes display no apparent associative interactions in *E. coli* similar to HEK293T despite the higher crowding in *E. coli*.

The in-cell interactions of negatively charged probes follow our wtBSA data, where a higher BSA concentration induced binding of the negatively charged probes but not the neutral ones. Indeed, previous work compared the nonspecific binding of various peptides and showed that neutral peptides such as polyserine were the most inert ones.^44,45^ If the comparison with wtBSA holds, then we can explain the binding of the negatively charged probes by higher crowding in *E. coli* (Supporting Discussion 2). However, given the high affinity of these glutamic acid-rich probes for highly cationic proteins, we cannot exclude specific binding to cationic proteins, metal ions, or polyamines.

It has been suggested that the net negative charge of the proteome ensures self-repulsion, enhancing protein solubility and mobility,^46^ while hydrophilicity also plays a role. We see that for our probes, the charge should be in a relatively neutral window for probes to be inert. A negative charge is thus not required to prevent interactions in the cytoplasm. These effects may be specific to serine, as this is a well-solvated and small amino acid. Together, we can conclude that nonspecific associative interactions are species-dependent.

### Stickiness upon ATP Depletion

Next, we perturbed the nonspecific binding landscape in HEK293T. ATP can interact with protein surfaces and reduce protein self-assembly.^47,48^ Moreover, ATP depletion and carbon starvation alter diffusion in *E. coli*(49,50) and baker’s yeast^51^ in a probe-dependent manner.^52−54^ We thus hypothesized that ATP changes the nonspecific interactions between the molecules in the cell. To test this, we treated HEK293T cells with 2 μM carbonyl cyanide-*p*-trifluoromethoxyphenylhydrazone (FCCP) to stop ATP synthesis,^55^ which is a condition in which most of its ATP gets depleted. We see that FCCP addition increases the ratio for most probes within 5 min, after which it stays the same or decreases (Figure 3D). Because the ratio of GFP(SSS)_4_ stays equal, we can exclude GFP-specific effects such as changes in water activity, viscosity, or pH effects (Figures S15 and S16). Direct ATP binding to the sensors can also be excluded because ATP does not bind the neutral or negatively charged sensors in a buffer and slightly decreases the ratio for cationic sensors (Figure S17), while in-cell ATP removal increases the ratio. The FCCP-induced ratiometric changes are thus due to interactions at the sensing domains.

We converted the ratios to the apparent percentage of the bound probe using the maximum binding affinity with BSA as a reference. This analysis shows that ATP depletion increases the apparent cationic/hydrophobic probe interactions and decreases the negative/hydrophilic ones (Figure 3E). These changes can be due to changes in the binding affinity or a change in the binding partner, which in either case means that the binding architecture in the cell changes upon ATP depletion. To confirm a change in binding behavior, we assessed binding with FRAP (Figure 3F and Figure S12), which showed an increase in the recovery time for GFP(KLL)_4_, verifying its altered bound state from the ratiometric readout. On the other hand, the GFP(KKK)_4_ already diffused slower and did not show an additional retardation, showing a more complex picture. The sparingly interacting GFP(ESE)_4_ and GFP(SSS)_4_ are at our lower FRAP detection limit and showed little change. The interaction change due to ATP depletion could have several sources. ATP has been shown to interact directly with proteins in vitro,^48,56,57^ which can disperse protein assemblies. However, other mechanisms, such as a response from the proteostasis network due to FCCP toxicity, cannot be excluded: The uncoupling of the mitochondrial membrane potential prevents the uptake of its precursor proteins that accumulate in the cytoplasm.^58^ Moreover, ATP hydrolysis can stimulate the release of clients from chaperones, and ATP depletion could result in irreversible complexes. Regardless of the precise mechanism, there is a difference in the interaction profile of the cytoplasm due to ATP depletion, which may contribute to experimental observations such as tracer-specific retardation of diffusion upon carbon depletion in yeast.^52^

## Conclusions

In the crowded cytoplasm, associative nonspecific interactions modulate the stability of the proteome, altering its organization and folding. To quantify these interactions in a crowded solution and inside cells, we demonstrate a method to monitor the hydrophobic and electrostatic contributions of protein interactions. We show that associative interactions of BSA solutions and cytoplasms of HEK293T and *E. coli* interact strongly with cationic and hydrophobic probes and not with hydrophilic neutral probes. However, highly anionic probes show a condition dependence where they are inert in HEK293T but not in *E. coli* or at high BSA concentrations. ATP depletion will further modulate this interaction architecture in the cells.

## Supporting Information Available

Supporting data on DNA sensing for sensor design, data, and control experiments accompanying Figures 1–3, supporting discussion on the probe development, supporting discussion on the role of activity and the Hill equation at high concentrations, and DNA sequences (PDF)
