# Supplementary material for: Probing Electrostatic and Hydrophobic Associative Interactions in Cells
Source: J Phys Chem B. 2024 Oct 30;128(44):10861–9. doi: 10.1021/acs.jpcb.4c05990 (PMC11551953; doi:10.1021/acs.jpcb.4c05990)
Supplement: Supplementary file 1 — jp4c05990_si_001.pdf [file jp4c05990_si_001.pdf]

Supporting Information

Probing Electrostatic and Hydrophobic Associative Interactions in Cells.

*Weiyan Zuo[2,3], Meng-Ruo Huang[2], Fabian Schmitz[1], Arnold J. Boersma[1,2]**

[1] Cellular Protein Chemistry, Bijvoet Centre for Biomolecular Research, Faculty of Science, Utrecht University, Utrecht, the Netherlands.

[2] DWI-Leibniz Institute for Interactive Materials, Forckenbeckstrasse 50, 52074 Aachen, NRW, Germany

[3] Institute of Technical and Macromolecular Chemistry, RWTH Aachen University, Worringerweg 1, 52074 Aachen, NRW, Germany

Supporting Discussion

Possible role of crowding in modulating the titration curves. The hydrophilic negatively charged probes display >20-30% binding at 100 mg/mL BSA, judging from the relative increase in ratio and comparison with complete binding. This is a relatively high BSA concentration: Timr et al. simulated that at 107 mg/mL, only 23% of the BSA is not in contact with other BSAs.(*1*) It is, therefore, highly likely that the peptide will be in a partially bound state. Given that BSA takes up much space at high concentrations, using the ligand concentration instead of its activity in the Hill equation becomes problematic. A formal analysis of the consequences of the BSA size would thus require the use of its activity. Instead of using the activity of BSA, we use a simple geometrical consideration, which merely serves to illustrate the response of binding under nonideal conditions.

In dilute conditions, assuming the Hill coefficient is 1, the fraction probe bound $\Theta = [L]/(K_d+[L])$, with [L] the BSA concentration and K_d the dissociation constant. The BSA concentration relates to the center-to-center distance *d* of the BSA molecules following $[L]\approx(1.18/d)^3$. However, the probe experiences the BSA surface, not its center of mass. Therefore, we take a simple geometric approach for illustrative purposes, where the peptide interacts with the BSA surface, and use the effective BSA concentration as $[L]_{eff} \approx(1.18/(d-2r))^3$, where *r* = the radius of BSA, which is 3.5 nm. The increased size would increase the collision rate, which correlates with the sum of the radii of the interacting particles, and we express this forward reaction rate change as an effective ligand concentration. Here, we assume that the diffusion coefficient of the molecules is unchanged as they travel through water until binding the nearest neighbor, and the peptide binds the entire sphere in an equal manner. Inserting $[L]_{eff}$ in the Hill equation using the K_d value from dilute conditions gives a BSA-size corrected fraction of bound peptide, Θ_{corr}. Plotting Θ_{corr} (that would be the output of the fluorescence measurements) versus [L] (as in the fluorescence experiments), gives curves with similar sigmoidal shapes as we see in our experiments for weak binders (**Figure below A-C**). When we fit these crowding-corrected binding curves with the Hill equation with the K_d and the Hill coefficient as free parameters, we see excellent fits where the Hill coefficient is well above 1 for weak binders and approaches 1 for tight binders under dilute conditions (**Figure below D**). This is the same as we see in our fluorescence experiments where weak binders such as EEE, ESS, and ESE have Hill coefficients of 2-3, whereas those of tight binders approach 1. In addition to the modified Hill coefficient, we see an overestimation of the binding coefficient for weak binders that require crowded conditions, while the BSA size does not affect the K_d estimation under dilute conditions (**Figure below E**). While this analysis is highly simplified by ignoring solute-solute interactions and assumes a single distance between all the surfaces, the trend is clear and the weak crowder binding probes can be simply described by considering the surface-to-surface distances instead of using the BSA concentration.

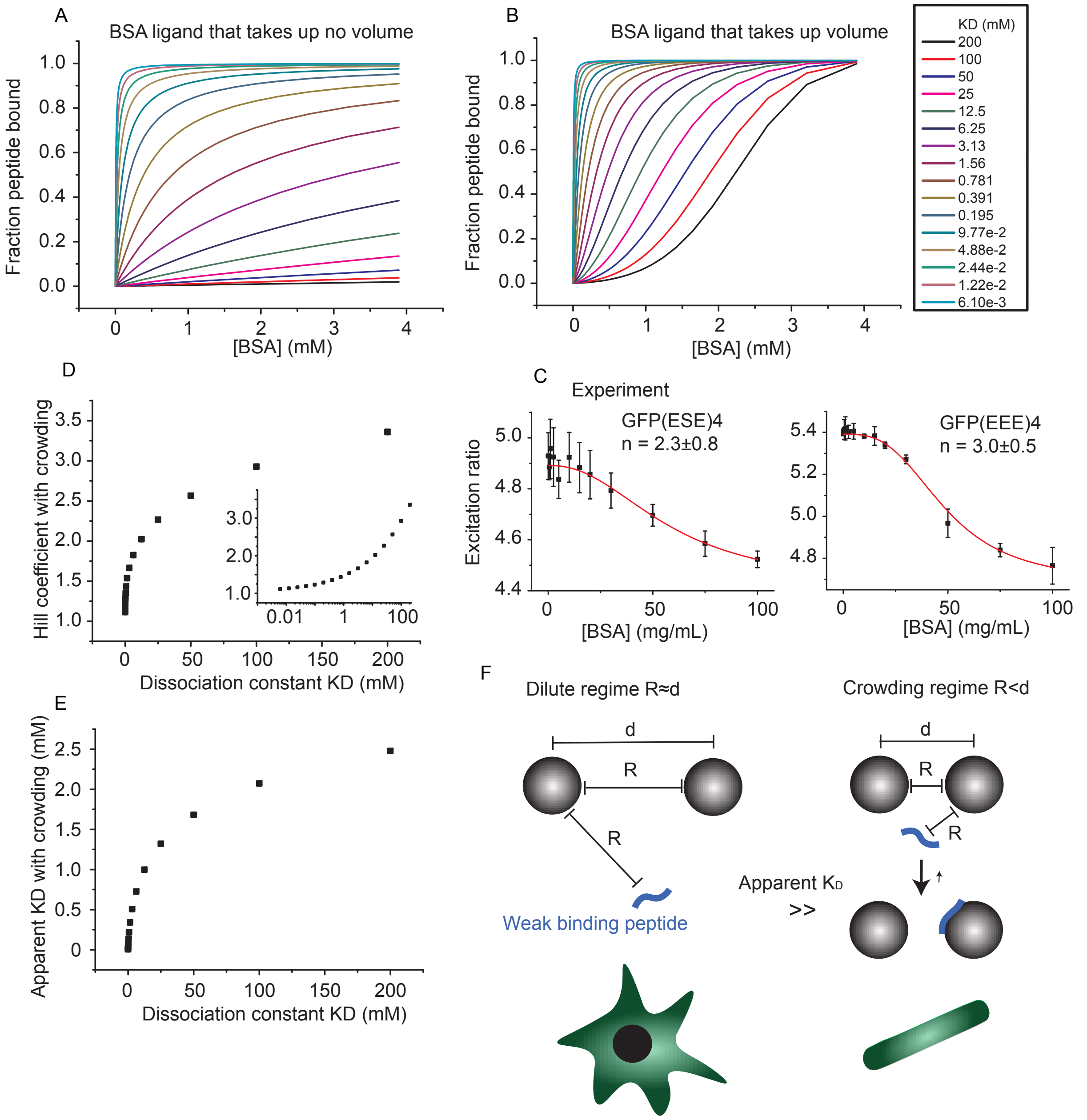

Figure Supporting discussion. Analysis of the consequence of the BSA size on the shape of the titration curve. A. Expected fraction peptide bound as calculated with the Hill equation with different dissociation constants where BSA does not occupy volume, and there is no cooperativity in binding. B. BSA reduces the distance between the molecules, altering the fraction peptide bound, which was plotted versus the BSA concentration for different dissociation constants, assuming no cooperativity in binding. C. Two example titration curves from our experimental data (black scatter) show the same sigmoidal-shaped binding curves for weak binding peptides. Depicted is the Hill coefficient obtained from the Hill equation (red line). D. Relation between the Hill coefficient obtained from fitting data in graph B to a Hill equation versus the dissociation constant. Weaker binders have a higher Hill coefficient. Inset is a logarithmic x-axis that shows the trend for tight binders. E. The binding affinity was obtained from fitting graph B to a Hill equation, showing an apparent K_D much higher than found under dilute conditions. F. Cartoon of the implications of the proposed effect of crowding. Depicted is the situation for the weak binders (e.g., GFP(EEE)$_4$, GFP(ESE)$_4$); left the dilute regime where the crowder size contributes less, right

the crowding regime where the binding is strongly enhanced by the increased crowder effective concentration. The dilute regime follows our observations in HEK293T, and the crowding regime that in *E. coli*.

Probe design and validation using a polynucleotide binding domain. To design a method to probe nonspecific associative interactions of the environment, we hypothesized the excitation spectrum of circular permuted GFP (cpGFP) would change when a fused interaction domain binds any component in the environment. This is comparable to the many excitation spectrum-based GFP sensors that measure the conformational change of a fused substrate-binding protein.(*2-5*) Their sensing principle is based on the perturbance of the GFP structure that alters the excitation spectrum. To design a probe that measures interactions with the environment, we started with the DNA-binding Lys-Trp-Lys repeat peptides $(KWK)_n$ because these have been well studied.(*6, 7*) We split the GFP backbone at Tyr145, which is near the chromophore, and the original N- and C- termini were connected with a flexible linker (GGSGGT), similar to previous cpGFP sensors.(*3*) We fused a single DNA sensing $(KWK)_4$ domain at the new N-terminus of the cpGFP, yielding cpGFP$(KWK)_4$. Titration of salmon testes DNA to cpGFP$(KWK)_4$ lead to an intensity decrease of the excitation spectrum for 515 nm emission (**Figure S1A**), showing that both the protonated (386 nm band) and deprotonated (472 nm band) forms of the chromophore are quenched. The 472 nm band reduces to ~46% of the original intensity and the 386 nm to ~76% of its original intensity. We quantify this change with the 405 nm/488 nm ratio as these can be directly compared to common laser excitation wavelengths (**Figure S1B**). The ratio increased until 0.1 mg/mL DNA, where the binding was complete. cpGFP without the KWK domains is not quenched by DNA (**Figure S1B**), indicating that DNA binds the peptide and not the cpGFP. To test the optimal site for peptide fusion, we fused the peptide at the N-termini of cpEGFPs introduced at Tyr145, Gln157, and Ala229. We saw that peptide insertion at the 145 and 229 positions generated similar quenching effects as cpGFP$(KWK)_4$, while the 157 position did not display quenching (**Figure S1D**). We thus continued with the Tyr145 and made a construct with $(KWK)_2$ domains fused to both the N- and C-terminus of cpEGFP at Tyr145 to assess if this would perturb the GFP more. However, this gave the same results as the corresponding $(KWK)_4$ construct (**Figure S1C**). To obtain higher sensitivity, we fused a $(KWK)_4$ domain at the N-terminus of cpEYFP. Although this construct gives much larger intensity changes upon DNA binding (**Figure S2A**), it is also highly KCl sensitive and was therefore discarded (**Figure S2B**). In contrast, the cpEGFP and cpGFP fusions were KCl insensitive (**Figure S4A**). We proceeded with the cpGFP$(KWK)_4$ construct because 1) cpGFP has a higher 405 nm excitation peak than cpEGFP, providing a more reliable ratiometric readout because it overcomes the higher autofluorescence of a cell at this wavelength, and 2) two separate $(XXX)_2$ peptides may induce intramolecular and divalency effects and thereby add complicating factors to understanding peptide binding.

As expected, cpGFP$(KWK)_4$ detects the chemically similar molecules RNA and ATP (**Figure S3**), albeit the latter requires ~1,000× higher concentration to see a similar ratio change. Furthermore, the pH has a low influence on the ratio (**Figure S4B**). As shown before for $(KWK)_n$ peptides,(*8*) the binding to

DNA was highly ionic strength sensitive (**Figure S4A**), further demonstrating the validity of the probing method. Hence, we have developed a new method to determine peptide interactions.

Table S1: Results from applying the Hill function to fit the BSA titration to the different sensors. The curves are depicted in Figure S7-9. Only the data was fit where a clear saturation curve was observed. [1)] The probe concentration is ~1 µM, and values close to this value are in the titration regime and therefore, the actual dissociation constant can be lower. Errors are errors in the fits. The fitting was not weighted for the errors in the individual data points; in that case, the Hill coefficient for GFP(KLL)$_4$ is, for example, 1.2±0.2 with a K_D of $(1.4 \pm 0.1) \times 10^2$ µM.

**GFP(XXX)$_4$**	**K_D (µM)**	**Hill coefficient**
	**wtBSA(-17e)**	
**KLL**	$(1.1 \pm 0.1) \times 10^2$	1.5±0.2
**KKK**	$(1.7 \pm 0.4) \times 10^2$	0.8±0.2
**ELL**	$(1.41 \pm 0.06) \times 10^2$	1.04±0.06
**KLK**	$(2.2 \pm 0.7) \times 10^2$	1.1±0.5
	**BSA(-74e)**	
**KLL[1)]**	1.2±0.4	0.8±0.1
**KSS**	$(2.0 \pm 0.5) \times 10^2$	1.6±0.4
**KSK**	13±3	1.4±0.4
**KLK**	11±4	0.8±0.2
**KKK[1)]**	1±2	0.4±0.2
**ELL**	$(2.4 \pm 0.9) \times 10^2$	1.6±0.6
	**BSA(+92e)**	
**ESS[1)]**	0.7±0.5	0.8±0.3
**EEE[1)]**	0.4±1.1	0.5±0.3
**ESE[1)]**	0.2±0.4	1.0±0.5
**KLL[1)]**	7±2	1.2±0.4

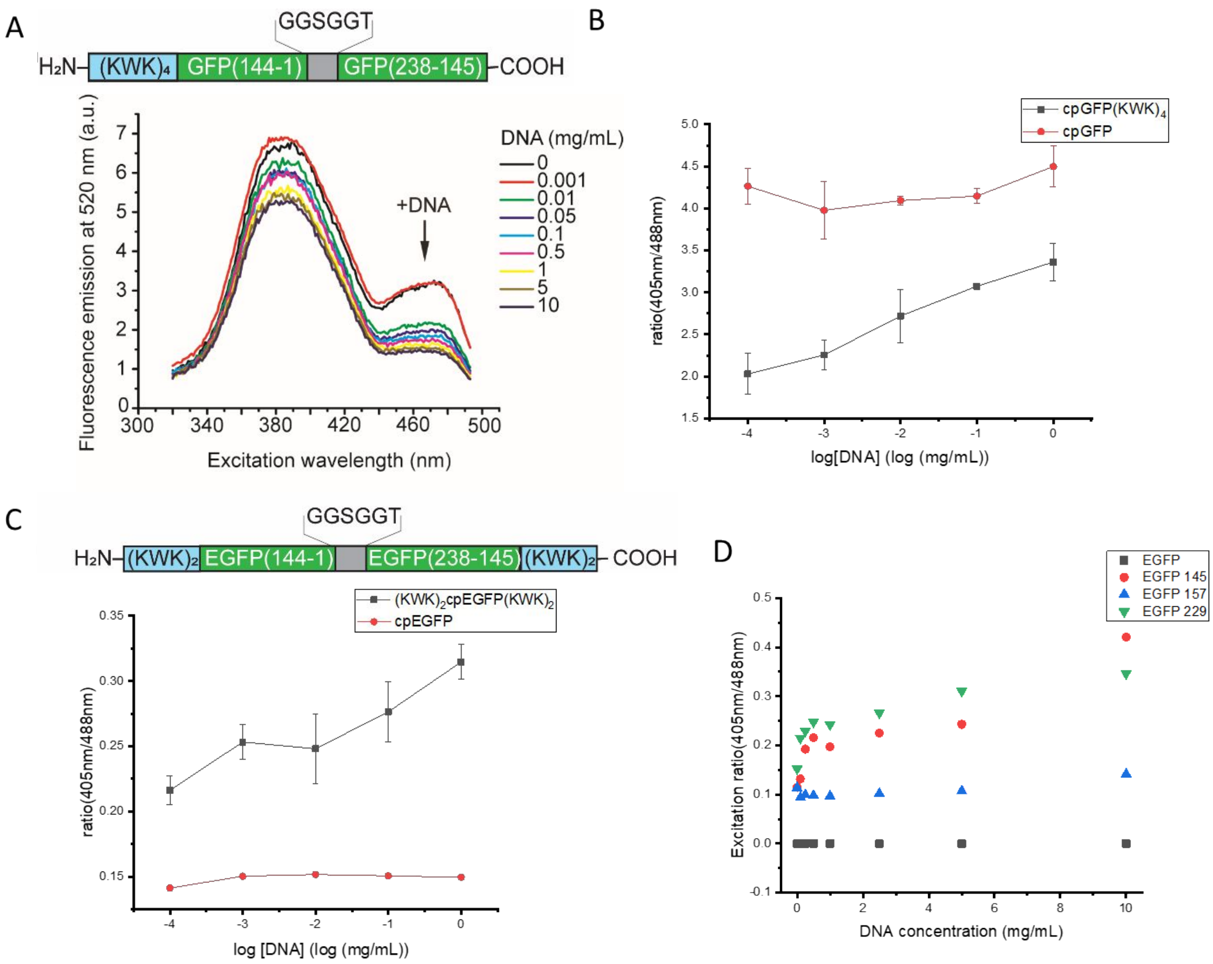

Figure S1: DNA titration to two different sensor designs followed by fluorometry. A. Excitation spectra (emission 520 nm) of cpGFP(KWK)$_4$ upon salmon testes DNA titration. B. Excitation ratio change (405 nm/488 nm) of cpGFP(KWK)$_4$ upon titrating DNA and comparison with the cpGFP control. C. Excitation ratio change (405 nm/488 nm) of (KWK)$_2$cpEGFP(KWK)$_2$ upon titrating DNA and compared with cpEGFP control. Buffer is 10 mM NaPi, pH 7.4. Data are the average of three independent experiments, and error bars are the corresponding standard deviations. D. Comparison between fusion sites for cpEGFP(KWK)$_4$ and EGFP control. DNA sensitivity is obtained for the 145 and 229 sites but not when the fusion site is located at 157.

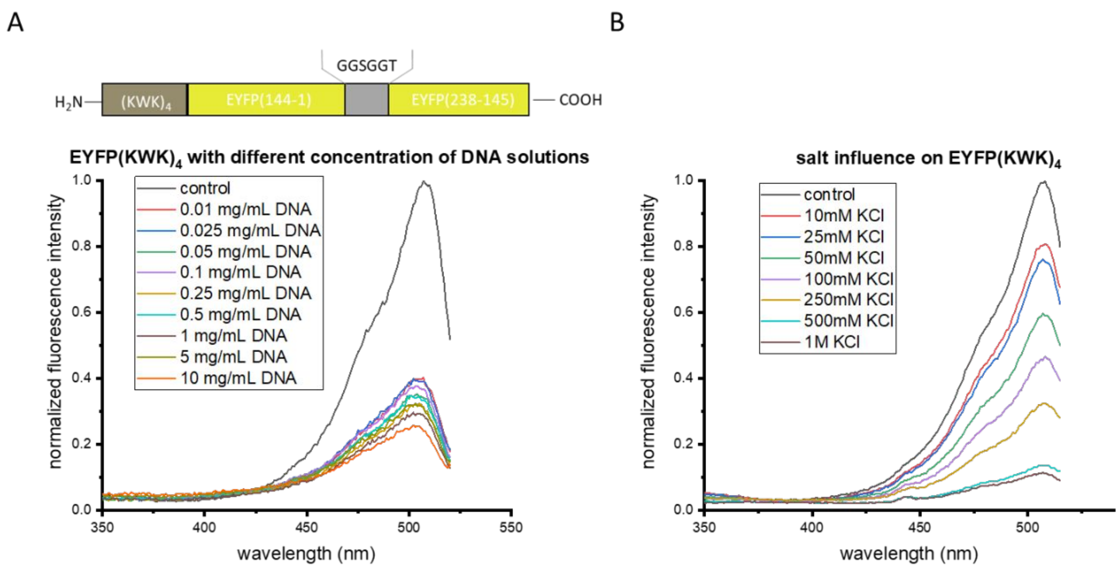

Figure S2: Salt sensitivity of EYFP(KWK)$_4$ measured by fluorescence plate reader. A. Normalized excitation spectra (emission 525 nm) of EYFP(KWK)$_4$ upon titrating st-DNA. B. Normalized excitation spectra of EYFP(KWK)$_4$ upon titrating KCl, showing a drastic decline in fluorescence. Buffer is 10 mM NaPi, pH 7.4.

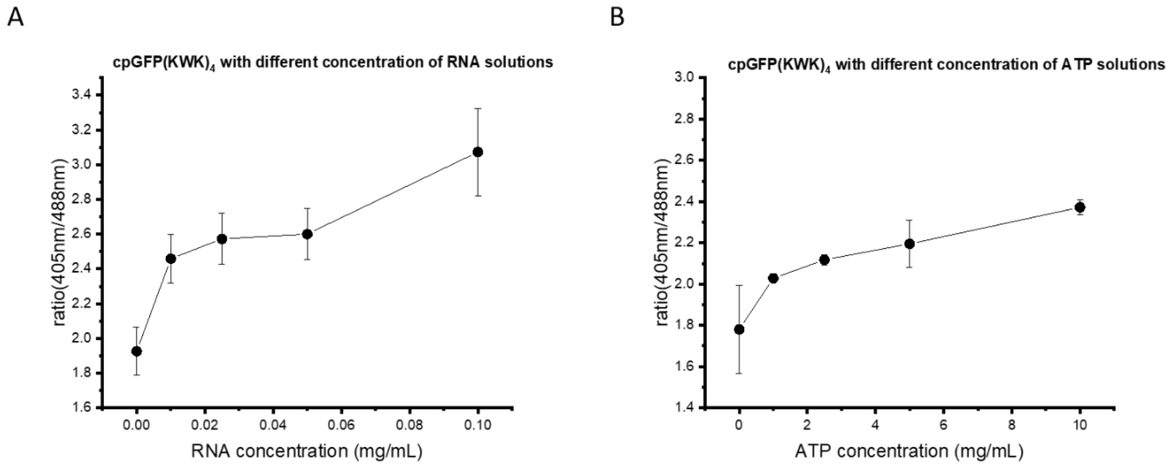

Figure S3. Titration of nucleic acids to cpGFP(KWK)$_4$ was monitored by fluorescence plate reader, showing the expected change in excitation ratio. A. Excitation ratio change of cpGFP(KWK)$_4$ upon RNA (from baker's yeast, Sigma-Aldrich) titration (stock pH 7.4). B. Ratio change of sensor cpGFP(KWK)$_4$ upon titrating ATP (stock pH 7.4). Buffer is 10 mM NaPi, pH 7.4. Data is the average of three independent experiments, and error bars are the corresponding standard deviations.

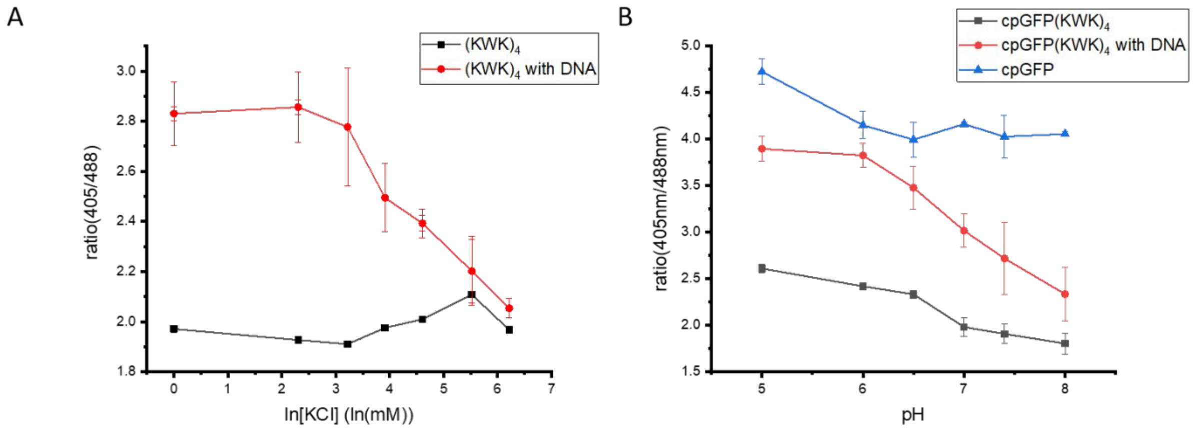

Figure S4. Salt and pH influence on cpGFP(KWK)$_4$ readout. A. Excitation ratio change of cpGFP(KWK)$_4$ (black) and cpGFP(KWK)$_4$ (red) with 0.1 mg/mL DNA solution upon KCl titration. The interaction between DNA and peptide was reduced by increasing [KCl]. B. pH dependence of the excitation ratio of cpGFP control (blue), cpGFP(KWK)$_4$ (black), and cpGFP(KWK)$_4$ with 0.1 mg/mL DNA (red) in 10 mM NaPi buffer.

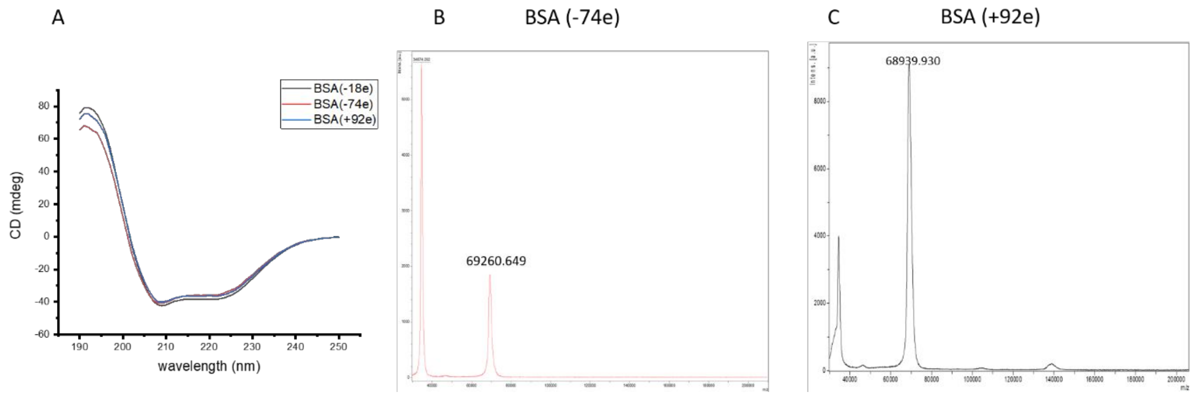

Figure S5. Characterization of surface-modified BSA. A. Assessment of secondary structure by circular dichroism CD spectra. 0.1 mg/mL BSA in 10mM NaPi buffer at 25 °C with an HT voltage lower than 500 V. B,C. MALDI-TOF mass spectrometry indicates the presence of the correctly modified BSA for BSA(-74e) and BSA(+92e). Super-DHB (Merck, Germany) was selected as the matrix. MALDI-TOF mass spectrometry was performed on an ultrafleXtreme Mass spectrometer (Bruker-Daltonics, Germany).

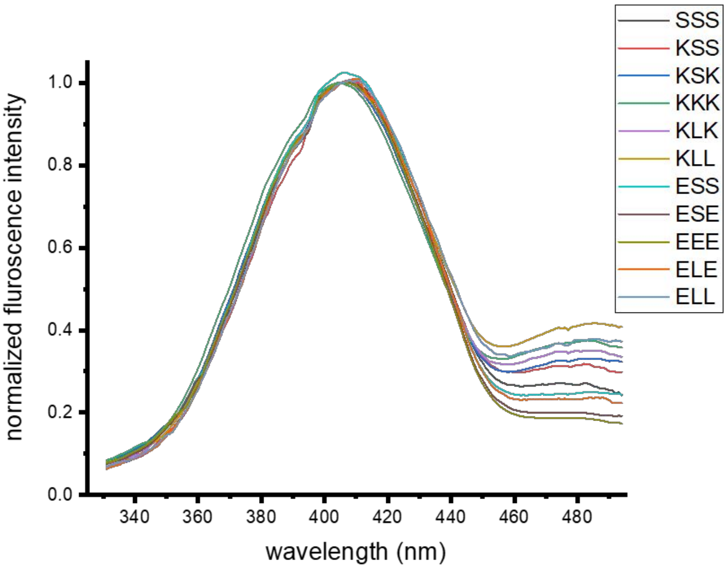

Figure S6. Normalized excitation spectra (emission 520 nm) for each of the sensors. All spectra were obtained in 10 mM NaPi, 100 mM KCl, and pH 7.4.

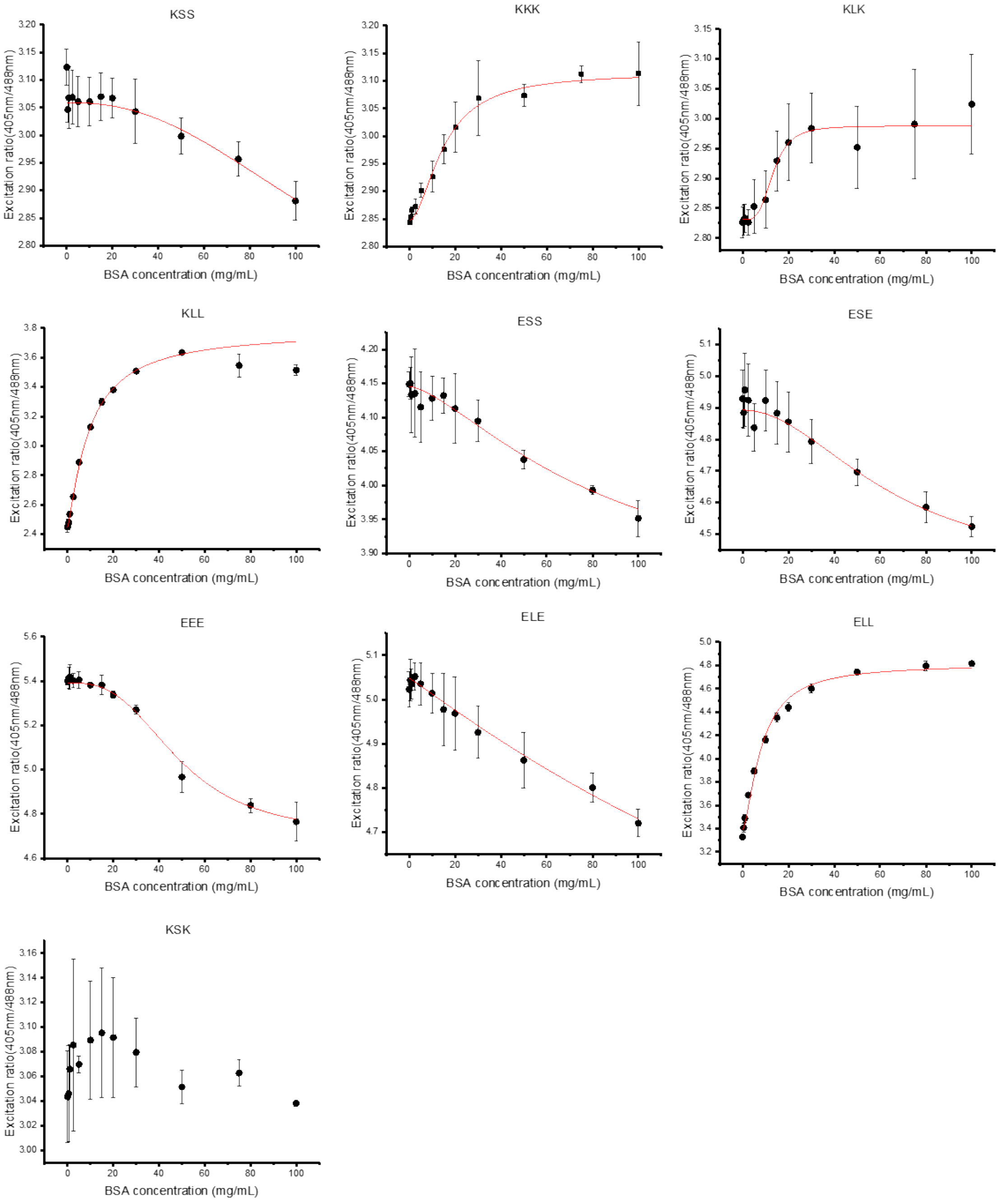

Figure S7. Dose-response curves for purified sensors upon wtBSA(-17e) titration. All data were obtained and analyzed as described in the methods section. Fits to the Hill function are shown, and results are displayed in **Table S1**. Probes that did not lead to saturated binding (GFP(XXX)$_4$ = ESS, ESE, EEE, ELE, KSK) were not further processed for dissociation constant determination. All experiments were repeated as triplicates, and error bars are standard deviations. Buffer conditions are described in the methods section. We note that 100 mg/mL protein is in the range of protein concentrations estimated for mammalian cell lines in cultures.(*9*)

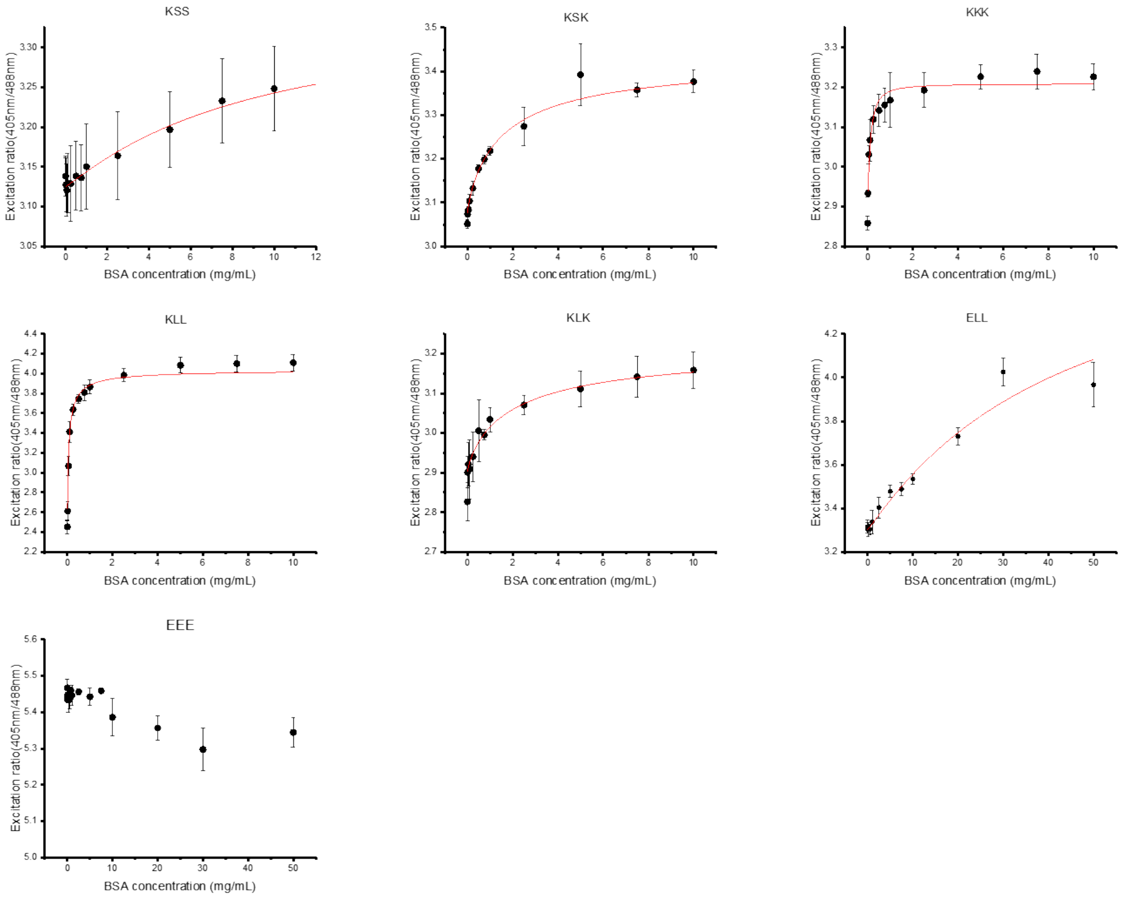

Figure S8. Dose-response curves for purified sensors upon BSA (-74e) titration. All data were obtained and analyzed as described in the methods section. Results are provided in **Table S1**. Excitation ratio changes of GFP(ESS)$_4$, GFP(ESE)$_4$, GFP(ELE)$_4$, and GFP(EEE)$_4$ did not show apparent saturation and were not processed for dissociation constant determination. All experiments were repeated as triplicates, and error bars are standard deviations. Buffer conditions are described in the methods section.

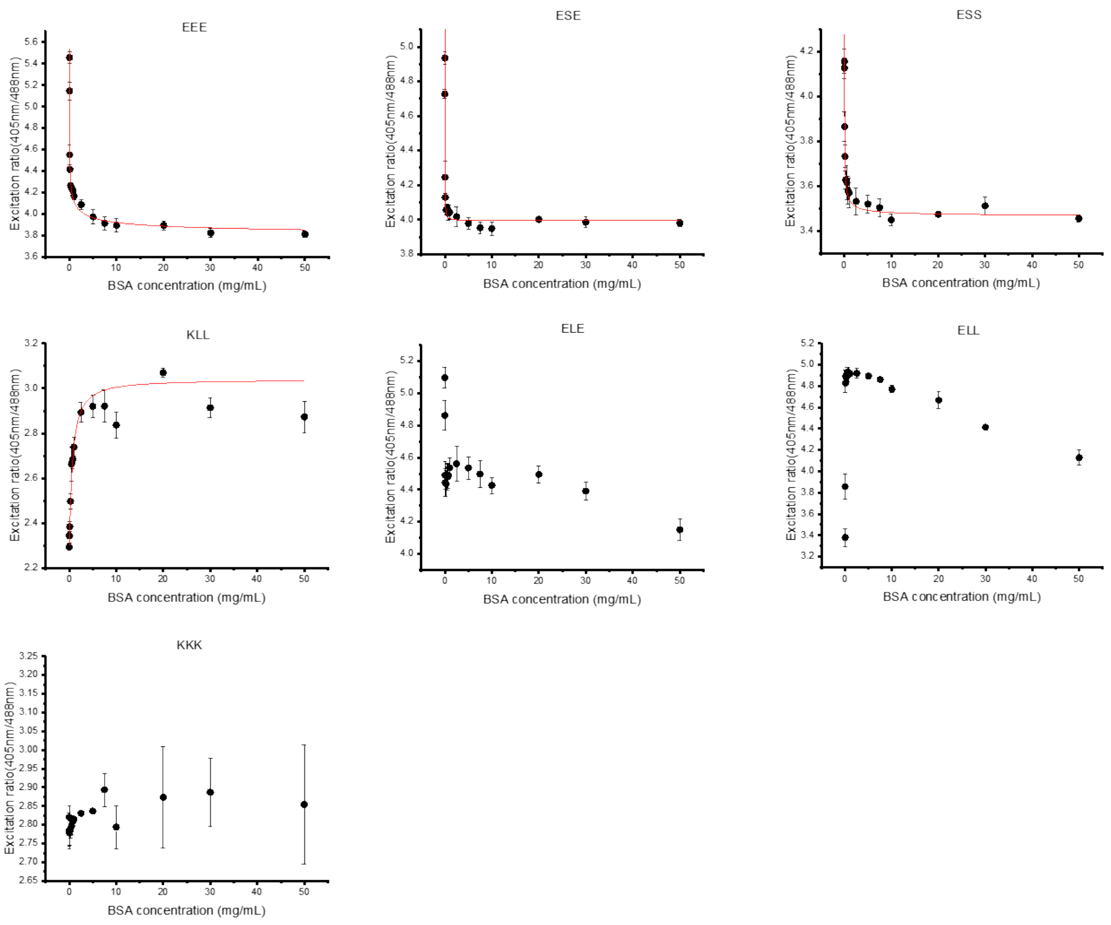

Figure S9. Dose-response curves for purified sensors upon BSA (+92e) titration. All data were obtained and analyzed as described in the methods section. The results are listed in **Table S1**. The ratiometric changes of GFP(KKK)$_4$, GFP(KSK)$_4$, GFP(KLK)$_4$, GFP(KSS)$_4$, GFP(ELE)$_4$, GFP(ELL)$_4$ were not fitted with a Hill function due to lack of saturation or more complex binding. All experiments were repeated as triplicates, and error bars are standard deviations. Buffer conditions are described in the methods section.

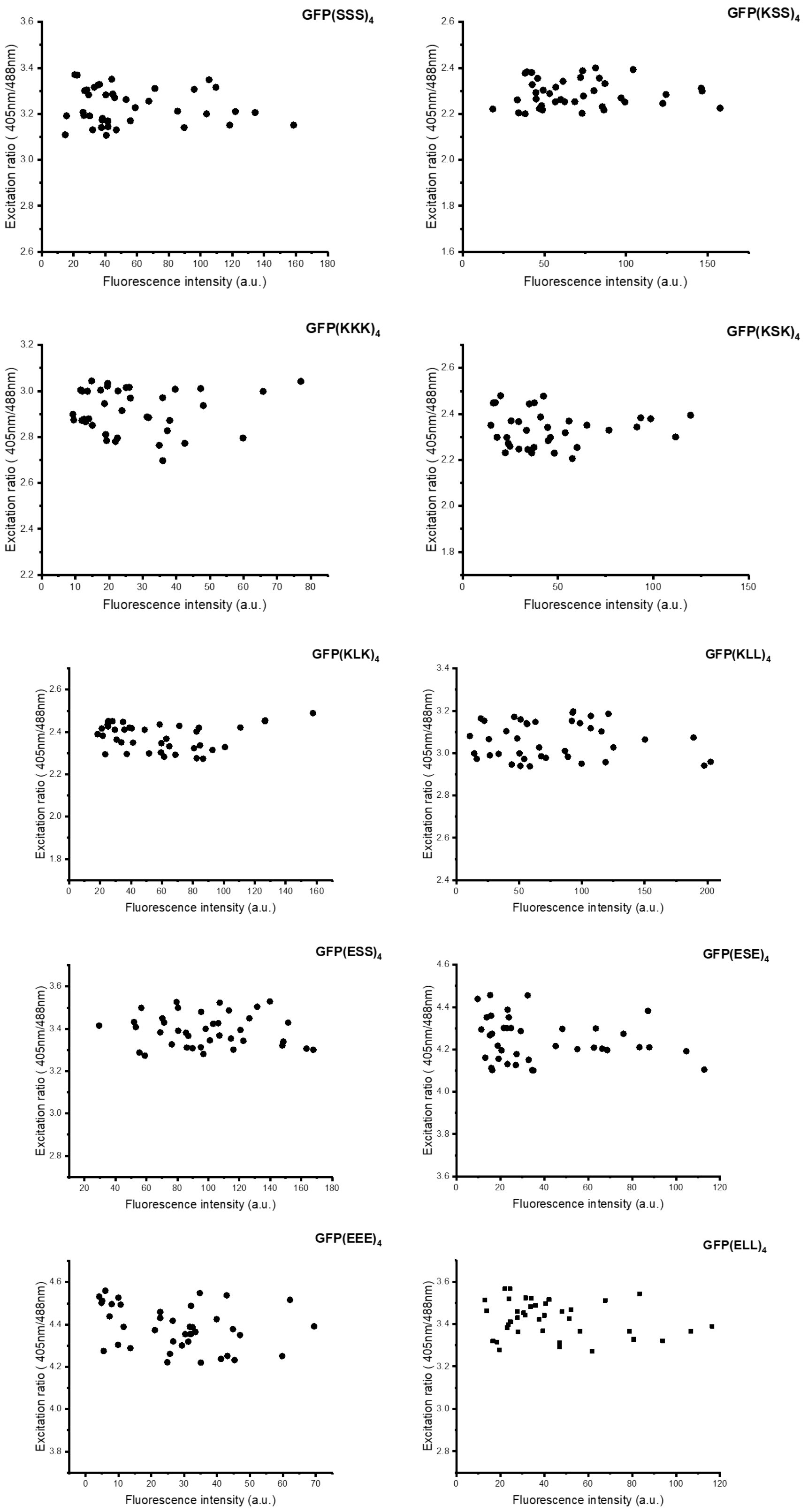

Figure S10. In-cell ratio showed no clear dependence on sensor concentration in HEK293T cells expressing GFP(XXX)$_4$. The excitation ratio (405nm/488nm) for each cell was plotted against its fluorescence intensity upon 405 nm excitation.

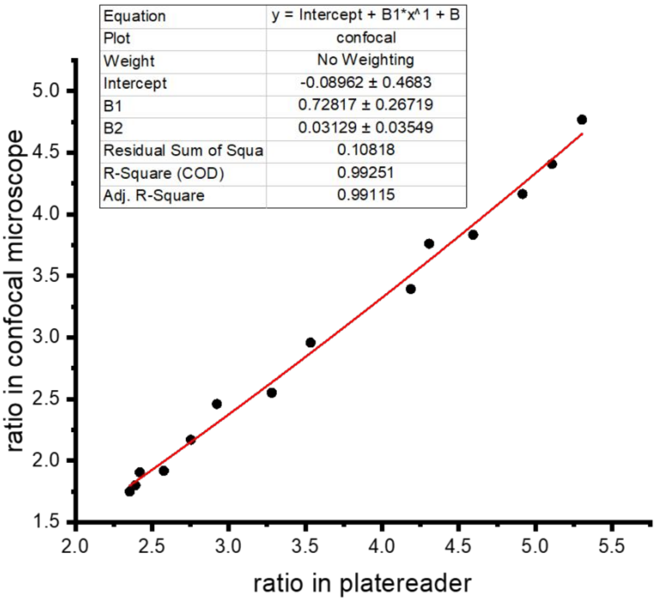

Figure S11. The empirical relation between 405/488 ratios determined by fluorescence plate reader and ratios determined by confocal microscope. Data points are the different sensors in buffer. A quadratic function was used to fit the data, allowing later conversion of ratios between the two devices.

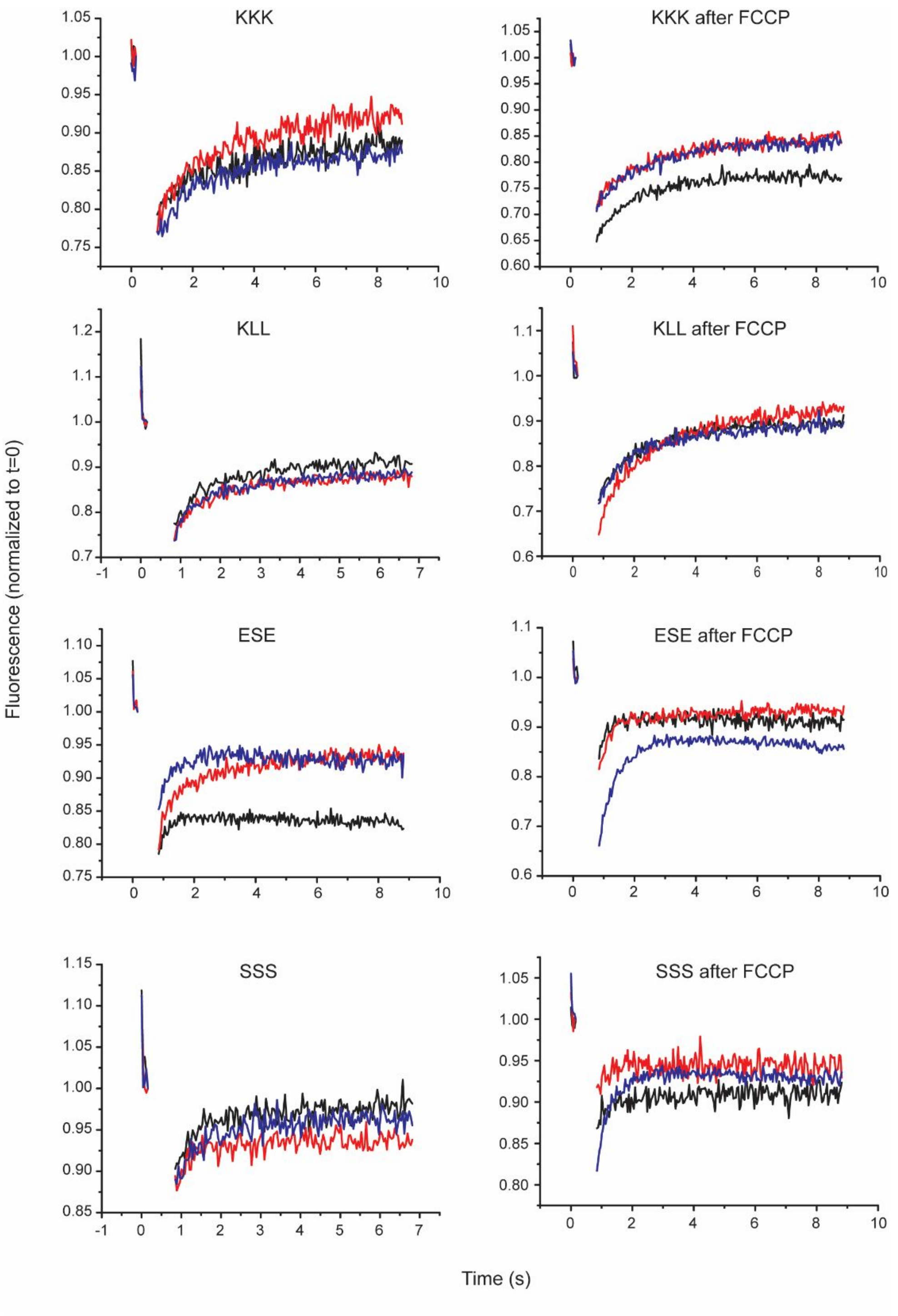

Figure S12. FRAP example traces. Three recovery traces for each probe after bleaching with 405 nm LED, showing the comparison between the probes and the variation between cells. The GFP(KKK)$_4$ and GFP(KLL)$_4$ (top two rows) recover slower than the GFP(ESE)$_4$ and GFP(SSS)$_4$ (bottom two rows). FRAP after FCCP is after 5 minutes of FCCP treatment.

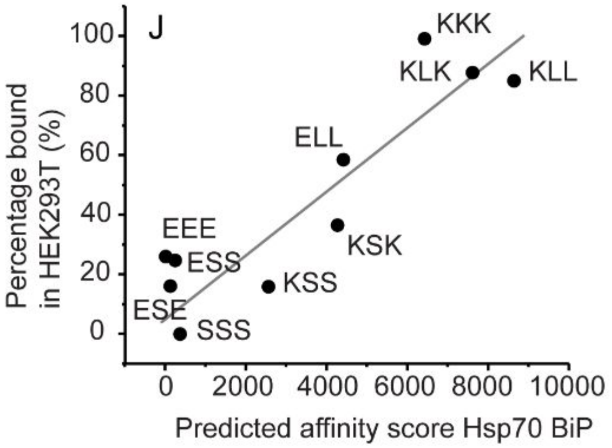

Figure S13. Determined binding percentages in HEK293T plotted versus predicted binding score to Hsp70 BiP in buffer (*10*). The line is to guide the eye.

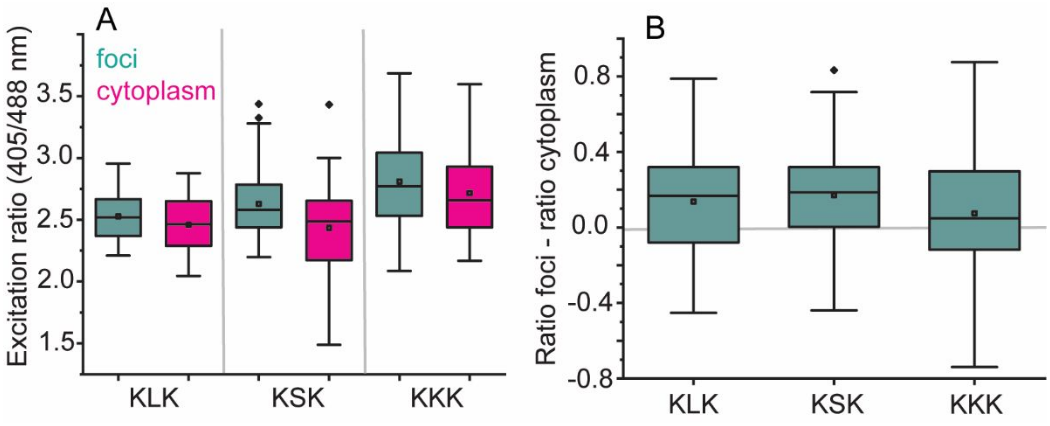

Figure S14. The ratiometric readout is higher in foci than the remainder of the cytoplasm of HEK293T. A. Average ratio of foci compared to average ratio of cytoplasms compared for the three probes that form foci. B. ratio difference per cell, showing a clear increase in ratio.

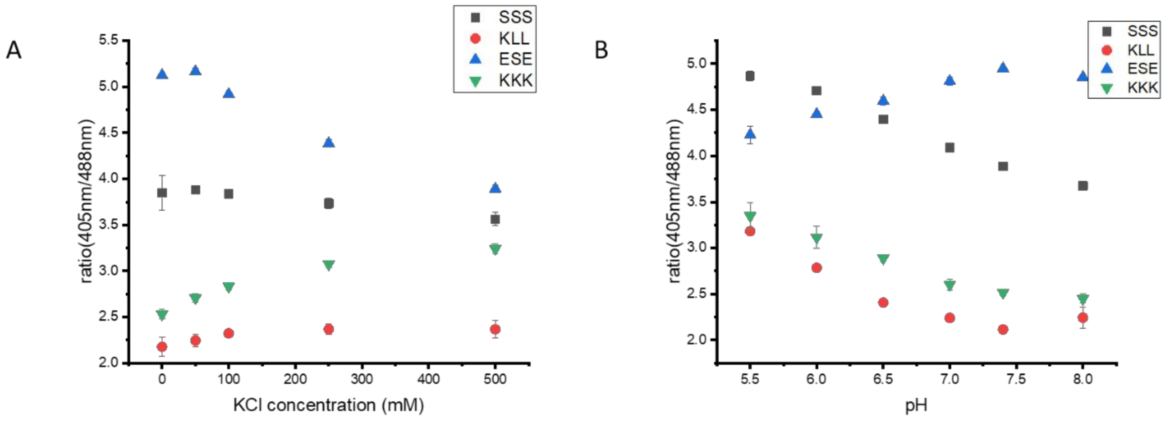

Figure S15. Salt and pH influence on the excitation ratio. A. Excitation ratio change of GFP(SSS)$_4$ (black), GFP(KLL)$_4$ (red), GFP(ESE)$_4$ (blue) and GFP(KKK)$_4$ (green) upon KCl titration. The sensitivity to KCl was sensor-dependent. B. Excitation ratio dependence on pH. The sensors are sensitive to the pH, which shows that readouts need to be monitored with a control sensor, GFP(SSS)$_4$. Note that in-cell measurements upon ATP depletion show no change in the GFP(SSS)$_4$ probe, and acidification can be excluded. All experiments were repeated as triplicates; error bars are standard deviations.

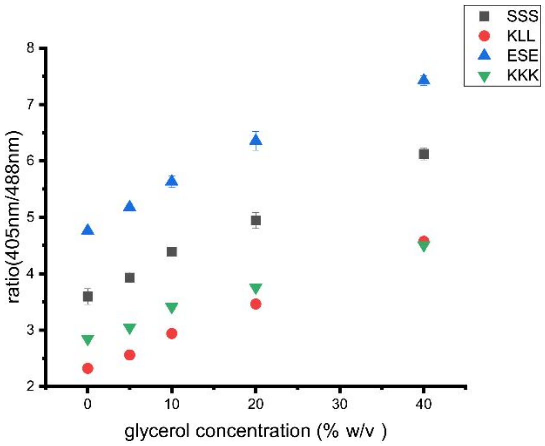

Figure S16. Glycerol titration to GFP(SSS)$_4$, GFP(KLL)$_4$, GFP(ESE)$_4$, and GFP(KKK)$_4$. Experiments in 10 mM NaPi pH 7.4. All sensors are sensitive to glycerol, possibly due to a change in water activity or viscosity. The ratio change is equal for the tested probes; therefore, if the need arises, allowing for correcting the ratios with the GFP(SSS)$_4$.

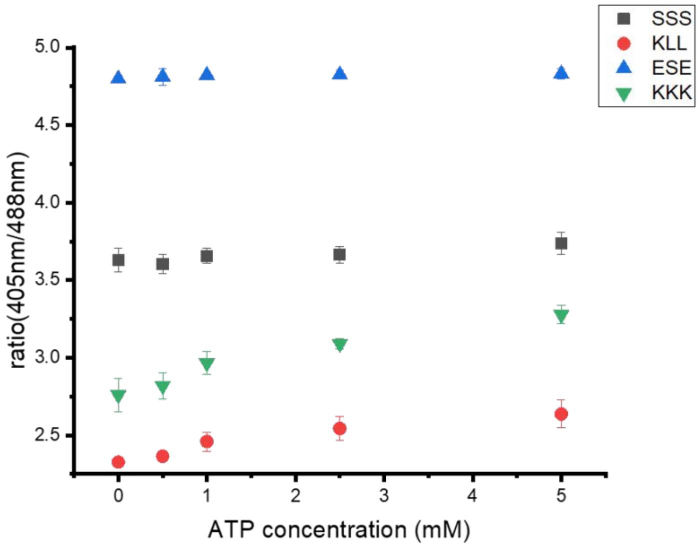

Figure S17. Sensitivity of purified sensor to the ATP concentration. GFP(SSS)$_4$ and GFP(ESE)$_4$ showed no excitation ratio change with ATP, while GFP(KLL)$_4$ and GFP(KKK)$_4$ showed ratio increases indicating that they bind ATP. Buffer is 10 mM NaPi, 100 mM KCl, pH 7.4. All experiments were repeated as triplicates; error bars are standard deviations.

Protein sequences.

The sequences for mammalian cell expression are shown. Those for overexpression in *E. coli* contain the MHHHHHHGSGENLYFQCG sequence at the N-terminus of the sequences below. In those cases, the first methionine in the sequences depicted below is omitted.

GFP(SSS)$_4$

MSSSSSSSSSSSSAFNSHNVYITADKQKNGIKVNFTVRHNVEDGSVQLADHYQQNTPIGDGPV
LLPDNHYLSTQTVLSKDPNEKRDHMVLHEYVNAAGITHGMDELYKGGSGGTMSKGEELFTG
VVPILVELDGDVNGHKFSVRGEGEGDATNGKLTLKFICTTGKLPVPWPTLVTTLSYGVQCFSR
YPDHMKQHDFFKSAMPEGYVQERTISFKDDGTYKTRAVVKFEGDTLVNRIELKGTDFKEDG
NILGHKLEYN

GFP(KSS)$_4$

MKSSKSSKSSKSSAFNSHNVYITADKQKNGIKVNFTVRHNVEDGSVQLADHYQQNTPIGDGP
VLLPDNHYLSTQTVLSKDPNEKRDHMVLHEYVNAAGITHGMDELYKGGSGGTMSKGEELFT
GVVPILVELDGDVNGHKFSVRGEGEGDATNGKLTLKFICTTGKLPVPWPTLVTTLSYGVQCFS
RYPDHMKQHDFFKSAMPEGYVQERTISFKDDGTYKTRAVVKFEGDTLVNRIELKGTDFKED
GNILGHKLEYN

GFP(KSK)$_4$

MKSKKSKKSKKSKAFNSHNVYITADKQKNGIKVNFTVRHNVEDGSVQLADHYQQNTPIGDG
PVLLPDNHYLSTQTVLSKDPNEKRDHMVLHEYVNAAGITHGMDELYKGGSGGTMSKGEELF
TGVVPILVELDGDVNGHKFSVRGEGEGDATNGKLTLKFICTTGKLPVPWPTLVTTLSYGVQC
FSRYPDHMKQHDFFKSAMPEGYVQERTISFKDDGTYKTRAVVKFEGDTLVNRIELKGTDFKE
DGNILGHKLEYN

GFP(KKK)$_4$

MKKKKKKKKKKKKAFNSHNVYITADKQKNGIKVNFTVRHNVEDGSVQLADHYQQNTPIGDG
PVLLPDNHYLSTQTVLSKDPNEKRDHMVLHEYVNAAGITHGMDELYKGGSGGTMSKGEELF
TGVVPILVELDGDVNGHKFSVRGEGEGDATNGKLTLKFICTTGKLPVPWPTLVTTLSYGVQC
FSRYPDHMKQHDFFKSAMPEGYVQERTISFKDDGTYKTRAVVKFEGDTLVNRIELKGTDFKE
DGNILGHKLEYN

GFP(KLK)$_4$

MKLKKLKKLKKLKAFNSHNVYITADKQKNGIKVNFTVRHNVEDGSVQLADHYQQNTPIGDG
PVLLPDNHYLSTQTVLSKDPNEKRDHMVLHEYVNAAGITHGMDELYKGGSGGTMSKGEELF
TGVVPILVELDGDVNGHKFSVRGEGEGDATNGKLTLKFICTTGKLPVPWPTLVTTLSYGVQC
FSRYPDHMKQHDFFKSAMPEGYVQERTISFKDDGTYKTRAVVKFEGDTLVNRIELKGTDFKE
DGNILGHKLEYN

GFP(KLL)$_4$

MKLLKLLKLLKLLAFNSHNVYITADKQKNGIKVNFTVRHNVEDGSVQLADHYQQNTPIGDG
PVLLPDNHYLSTQTVLSKDPNEKRDHMVLHEYVNAAGITHGMDELYKGGSGGTMSKGEELF
TGVVPILVELDGDVNGHKFSVRGEGEGDATNGKLTLKFICTTGKLPVPWPTLVTTLSYGVQC
FSRYPDHMKQHDFFKSAMPEGYVQERTISFKDDGTYKTRAVVKFEGDTLVNRIELKGTDFKE
DGNILGHKLEYN

GFP(ELL)$_4$

MELLELLELLELLAFNSHNVYITADKQKNGIKVNFTVRHNVEDGSVQLADHYQQNTPIGDGP
VLLPDNHYLSTQTVLSKDPNEKRDHMVLHEYVNAAGITHGMDELYKGGSGGTMSKGEELFT

GVVPILVELDGDVNGHKFSVRGEGEGDATNGKLTLKFICTTGKLPVPWPTLVTTLSYGVQCFS
RYPDHMKQHDFFKSAMPEGYVQERTISFKDDGTYKTRAVVKFEGDTLVNRIELKGTDFKED
GNILGHKLEYN

GFP(ELE)$_4$

MELEELEELEELEAFNSHNVYITADKQKNGIKVNFTVRHNVEDGSVQLADHYQQNTPIGDGP
VLLPDNHYLSTQTVLSKDPNEKRDHMVLHEYVNAAGITHGMDELYKGGSGGTMSKGEELFT
GVVPILVELDGDVNGHKFSVRGEGEGDATNGKLTLKFICTTGKLPVPWPTLVTTLSYGVQCFS
RYPDHMKQHDFFKSAMPEGYVQERTISFKDDGTYKTRAVVKFEGDTLVNRIELKGTDFKED
GNILGHKLEYN

GFP(EEE)$_4$

MEEEEEEEEEEEEAFNSHNVYITADKQKNGIKVNFTVRHNVEDGSVQLADHYQQNTPIGDGP
VLLPDNHYLSTQTVLSKDPNEKRDHMVLHEYVNAAGITHGMDELYKGGSGGTMSKGEELFT
GVVPILVELDGDVNGHKFSVRGEGEGDATNGKLTLKFICTTGKLPVPWPTLVTTLSYGVQCFS
RYPDHMKQHDFFKSAMPEGYVQERTISFKDDGTYKTRAVVKFEGDTLVNRIELKGTDFKED
GNILGHKLEYN

GFP(ESE)$_4$

MESEESEESEESEAFNSHNVYITADKQKNGIKVNFTVRHNVEDGSVQLADHYQQNTPIGDGP
VLLPDNHYLSTQTVLSKDPNEKRDHMVLHEYVNAAGITHGMDELYKGGSGGTMSKGEELFT
GVVPILVELDGDVNGHKFSVRGEGEGDATNGKLTLKFICTTGKLPVPWPTLVTTLSYGVQCFS
RYPDHMKQHDFFKSAMPEGYVQERTISFKDDGTYKTRAVVKFEGDTLVNRIELKGTDFKED
GNILGHKLEYN

GFP(ESS)$_4$

MESSESSESSESSAFNSHNVYITADKQKNGIKVNFTVRHNVEDGSVQLADHYQQNTPIGDGPV
LLPDNHYLSTQTVLSKDPNEKRDHMVLHEYVNAAGITHGMDELYKGGSGGTMSKGEELFTG
VVPILVELDGDVNGHKFSVRGEGEGDATNGKLTLKFICTTGKLPVPWPTLVTTLSYGVQCFSR
YPDHMKQHDFFKSAMPEGYVQERTISFKDDGTYKTRAVVKFEGDTLVNRIELKGTDFKEDG
NILGHKLEYN

References

1. S. Timr, S. Melchionna, P. Derreumaux, F. Sterpone, Optimized OPEP Force Field for Simulation of Crowded Protein Solutions. *J Phys Chem B* **127**, 3616-3623 (2023).
2. G. S. Baird, D. A. Zacharias, R. Y. Tsien, Circular permutation and receptor insertion within green fluorescent proteins. *Proc Natl Acad Sci U S A* **96**, 11241-11246 (1999).
3. J. Akerboom *et al.*, Crystal structures of the GCaMP calcium sensor reveal the mechanism of fluorescence signal change and aid rational design. *J Biol Chem* **284**, 6455-6464 (2009).
4. T. Ichimura, H. Fujita, K. Yoshizawa, T. M. Watanabe, Engineering strain-sensitive yellow fluorescent protein. *Chemical Communications* **48**, 7871-7873 (2012).
5. R. Y. Tsien, The green fluorescent protein. *Annual review of biochemistry* **67**, 509-544 (1998).
6. S. Lee *et al.*, DNA binding fluorescent proteins for the direct visualization of large DNA molecules. *Nucleic Acids Res* **44**, e6 (2016).
7. D. P. Mascotti, T. M. Lohman, Thermodynamics of single-stranded RNA and DNA interactions with oligolysines containing tryptophan. Effects of base composition. *Biochemistry* **32**, 10568-10579 (1993).
8. J. D. Ballin, I. A. Shkel, M. T. Record, Jr., Interactions of the KWK6 cationic peptide with short nucleic acid oligomers: demonstration of large Coulombic end effects on binding at 0.1-0.2 M salt. *Nucleic Acids Res* **32**, 3271-3281 (2004).
9. B. Monterroso *et al.*, Macromolecular Crowding, Phase Separation, and Homeostasis in the Orchestration of Bacterial Cellular Functions. *Chem Rev* **124**, 1899-1949 (2024).
10. M. Schneider *et al.*, BiPPred: Combined sequence-and structure-based prediction of peptide binding to the Hsp70 chaperone BiP. *Proteins: Structure, Function, and Bioinformatics* **84**, 1390-1407 (2016).
